# A functional and structural comparative analysis of large tumor antigens reveals evolution of different importin α‐dependent nuclear localization signals

**DOI:** 10.1002/pro.4876

**Published:** 2024-02-01

**Authors:** Emily M. Cross, Nasim Akbari, Hanieh Ghassabian, Mikayla Hoad, Silvia Pavan, Daryl Ariawan, Camilla M. Donnelly, Enrico Lavezzo, Gayle F. Petersen, Jade K. Forwood, Gualtiero Alvisi

**Affiliations:** ^1^ School of Dentistry and Medical Sciences Charles Sturt University Wagga Wagga Australia; ^2^ Diamond Light Source Harwell Science and Innovation Campus Didcot United Kingdom; ^3^ Department of Molecular Medicine University of Padova Padova Italy; ^4^ Dementia Research Centre Macquarie University Sydney Australia; ^5^ Gulbali Institute Charles Sturt University Wagga Wagga Australia

**Keywords:** cNLS, importin alpha structure, importins, large T antigens, Merkel cell polyomavirus, NLS, NLS evolution, nuclear transport, oncogenes

## Abstract

Nucleocytoplasmic transport regulates the passage of proteins between the nucleus and cytoplasm. In the best characterized pathway, importin (IMP) α bridges cargoes bearing basic, classical nuclear localization signals (cNLSs) to IMPβ1, which mediates transport through the nuclear pore complex. IMPα recognizes three types of cNLSs via two binding sites: the major binding site accommodates monopartite cNLSs, the minor binding site recognizes atypical cNLSs, while bipartite cNLSs simultaneously interact with both major and minor sites. Despite the growing knowledge regarding IMPα‐cNLS interactions, our understanding of the evolution of cNLSs is limited. We combined bioinformatic, biochemical, functional, and structural approaches to study this phenomenon, using polyomaviruses (PyVs) large tumor antigens (LTAs) as a model. We characterized functional cNLSs from all human (H)PyV LTAs, located between the LXCXE motif and origin binding domain. Surprisingly, the prototypical SV40 monopartite NLS is not well conserved; HPyV LTA NLSs are extremely heterogenous in terms of structural organization, IMPα isoform binding, and nuclear targeting abilities, thus influencing the nuclear accumulation properties of full‐length proteins. While several LTAs possess bipartite cNLSs, merkel cell PyV contains a hybrid bipartite cNLS whose upstream stretch of basic amino acids can function as an atypical cNLS, specifically binding to the IMPα minor site upon deletion of the downstream amino acids after viral integration in the host genome. Therefore, duplication of a monopartite cNLS and subsequent accumulation of point mutations, optimizing interaction with distinct IMPα binding sites, led to the evolution of bipartite and atypical NLSs binding at the minor site.

## 1 INTRODUCTION

**FIGURE 1:**
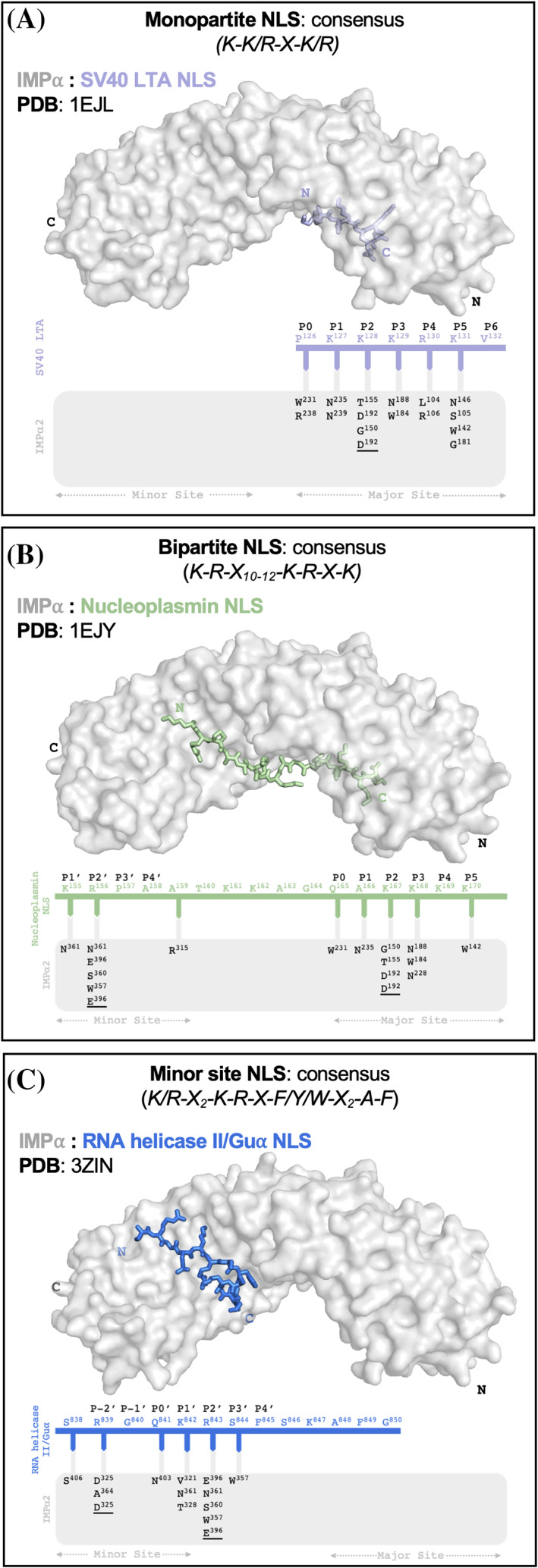
Structural determinants of IMP⍺‐dependent cNLSs and distinct NLS consensuses. Crystal structures of the prototypical SV40 LTA monopartite cNLS (PDB: 1EJL) (A), the nucleoplasmin bipartite cNLS (PDB: 1EJY) (B), and the RNA helicase II/Gua minor site‐specific NLS (PDB: 3ZIN) (C), each bound to mouse IMP⍺2ΔIBB. Gray cartoon represents IMP⍺2ΔIBB and colored sticks represent the cNLS peptides. Schematic representations of the binding interactions are shown below each structure, detailing hydrogen bonds and salt bridges (underlined). The minor site peptide in the SV40 LTA cNLS structure and major site peptide in the RNA helicase II/Gua structure were omitted for this figure. Pymol was used to generate figures and PDBePISA was used for all binding interaction calculations. cNLS, classical nuclear localization signal; NLS, nuclear localization signal.

Facilitated nuclear import of molecules is an active and signal‐dependent process, mediated by cellular transporters belonging to the importin (IMP) β superfamily (Alvisi et al., 2013). IMPβ1, or one of its 20 homologs, recognizes specific nuclear localization signals (NLSs) on cargo before translocating the complex across the nuclear envelope through the aqueous channel formed by the nuclear pore complex (NPC) and by direct interaction with hydrophobic nucleoporins (Kimura & Imamoto, 2014). Once within the nucleus, binding of Ran‐GTP to IMPβs induces a conformational change triggering complex dissociation and cargo release before recycling transporters back to the cytosol for new rounds of import. The first NLS was described in Simian Virus 40 (SV40) large tumor antigen (LTA) as a basic stretch of amino acids (aas) (126‐P**KKKRK**V‐132) sufficient and necessary for protein nuclear import (Kalderon, Richardson, et al., 1984; Kalderon, Roberts, et al., 1984). LTA nuclear import is strictly required for viral replication, since substitutions abolishing nuclear targeting without affecting any additional LTA biochemical properties hamper viral replication (Kalderon, Richardson, et al., 1984; Lanford et al., 1985; Lanford & Butel, 1980a, 1984; Paucha et al., 1985). Since its discovery, the role of SV40‐NLS in nuclear transport has been extensively characterized from a biochemical, structural, and functional point of view, rapidly becoming the prototype of the now so‐called monopartite classical (c)NLS (Conti et al., 1998; Hodel et al., 2001, 2006). In such pathway, IMPβ1 recognizes cNLS‐bearing cargoes through the adapter protein IMPα. Humans possess seven IMPα isoforms (IMPα1, 3–8), which are specifically expressed in different tissues and developmental stages and are endowed with specific cNLS recognition abilities (Miyamoto et al., 2016; Ninpan et al., 2016; Pumroy et al., 2015). The N‐terminus of IMPα features a c. 40 aa long, highly basic importin beta binding (IBB) domain. When not complexed with IMPβ1, the IBB domain competes with cNLSs for interaction with two NLS bindings sites on IMPα, thus playing an autoinhibitory role (Kobe, 1999). IMPα‐NLS binding sites are located in the armadillo (ARM) domain, formed by repetition of three organized α‐helices known as “ARM repeats.” ARM repeats create a minor and a major NLS binding site on the inner concave region of IMPα, allowing high‐affinity interactions with two or five basic residues of the cNLS, respectively (Conti et al., 1998). Monopartite cNLSs, such as that described for SV40 LTA, functionally interact with the IMPα major binding site. For some proteins however, when binding at the major site is suboptimal, a further interaction of the IMPα minor binding site with an additional basic stretch of aa located typically 10–12 aas upstream is required, thus forming a bipartite NLS which simultaneously interacts with both binding sites (Dingwall et al., 1987; Fontes et al., 2000). A novel yet still poorly characterized type of cNLS was recently found to selectively bind to the IMPα minor binding site and dubbed an atypical or type 3 cNLS (Kosugi, Hasebe, Matsumura, et al., 2009). While both monopartite and bipartite NLSs bind to IMPα in an extended conformation (Fontes et al., 2000), minor site‐specific NLSs use a distinct binding mode featuring an α‐helix at the C‐terminus of the NLS (Chang et al., 2013). Extensive biochemical, structural, and functional characterizations of IMPα interactions with such NLS types have allowed definition of distinct NLS consensuses (Figure 1), and the identification of a great number of nuclear proteins in the last 40 years (Chang et al., 2013; Christie et al., 2015; Fontes et al., 2000). However, little is known about the evolution of these three different types of IMPα‐dependent NLSs. Here we combined bioinformatics, biochemical, functional, and structural approaches to study this phenomenon using polyomaviruses (PyVs) LTAs as a model, which we believe represent an ideal starting point to study NLS evolution. As LTAs need to be translocated into the nucleus to participate in viral genome replication, all must contain a functional NLS (Lanford & Butel, 1984). Other than studies on SV40, NLSs have been identified for only two additional LTAs: in mouse PyV A2 (MPyV), two cNLSs have been described: one in an analogous position to SV40 (280‐P**KK**A**R**ED‐286) and one located approximately 100 aas upstream (189‐VS**RKR**P**R**PA‐197) (Richardson et al., 1986); in Merkel Cell PyV (MCPyV), a monopartite cNLS (275‐PFS**RKRK**‐280) is believed to be responsible for nuclear import (Nakamura et al., 2010). *Polyomaviridae* is a large and ancient viral family comprising more than 100 viral species which infect mammals, fishes, birds, and possibly even insects (Torres, 2020), which have been evolving for approximatively 500 million years (DeCaprio & Garcea, 2013). Therefore, it is reasonable to expect all LTAs from different PyVs to localize into the cell nucleus, possibly using different NLSs. Our analysis revealed that each human PyV (HPyV) LTA bears at least one functional cNLS, located between the LXCXE motif, which binds to the retinoblastoma (Rb) protein (Borchert et al., 2014), and the origin binding domain (OBD), in a position similar to that originally described for SV40 LTA (Kalderon, Roberts, et al., 1984). However, their structural organization as well as their IMPα binding and nuclear targeting abilities are extremely heterogenous. Indeed, while some LTAs contain a single monopartite cNLS, others, including Saint Louis (STL) and Karolinska Institute (KI) PyV LTAs, contain bipartite cNLSs, resulting in stronger IMPα binding and nuclear targeting abilities. Intriguingly and in contrast with current knowledge (Nakamura et al., 2010), MCPyV LTA contains an additional cNLS downstream of the one previously identified, forming a hybrid NLS. The latter normally functions as a bipartite cNLS, but, following deletion of the downstream basic aas upon viral integration in the host chromosomes, it can bind to the IMPα minor binding site alone. Bioinformatic analysis of LTAs from 115 deposited sequences revealed that a very high percentage of LTAs possess a cNLS located between the LXCXE motif and the OBD. The most notable exception to this is represented by PyVs infecting birds, where a cNLS is present in only 50% of analyzed LTAs, raising the possibility of an alternative import pathway to IMPα/β. Furthermore, our analysis showed that the cNLS is bipartite in almost 50% of cases, suggesting that bipartite NLSs could possibly have originated by multiple independent duplication events of an ancestral monopartite cNLS during evolution of PyVs.

## 2 RESULTS

### 2.1 Identification of cNLSs in HPyV LTAs

**FIGURE 2:**
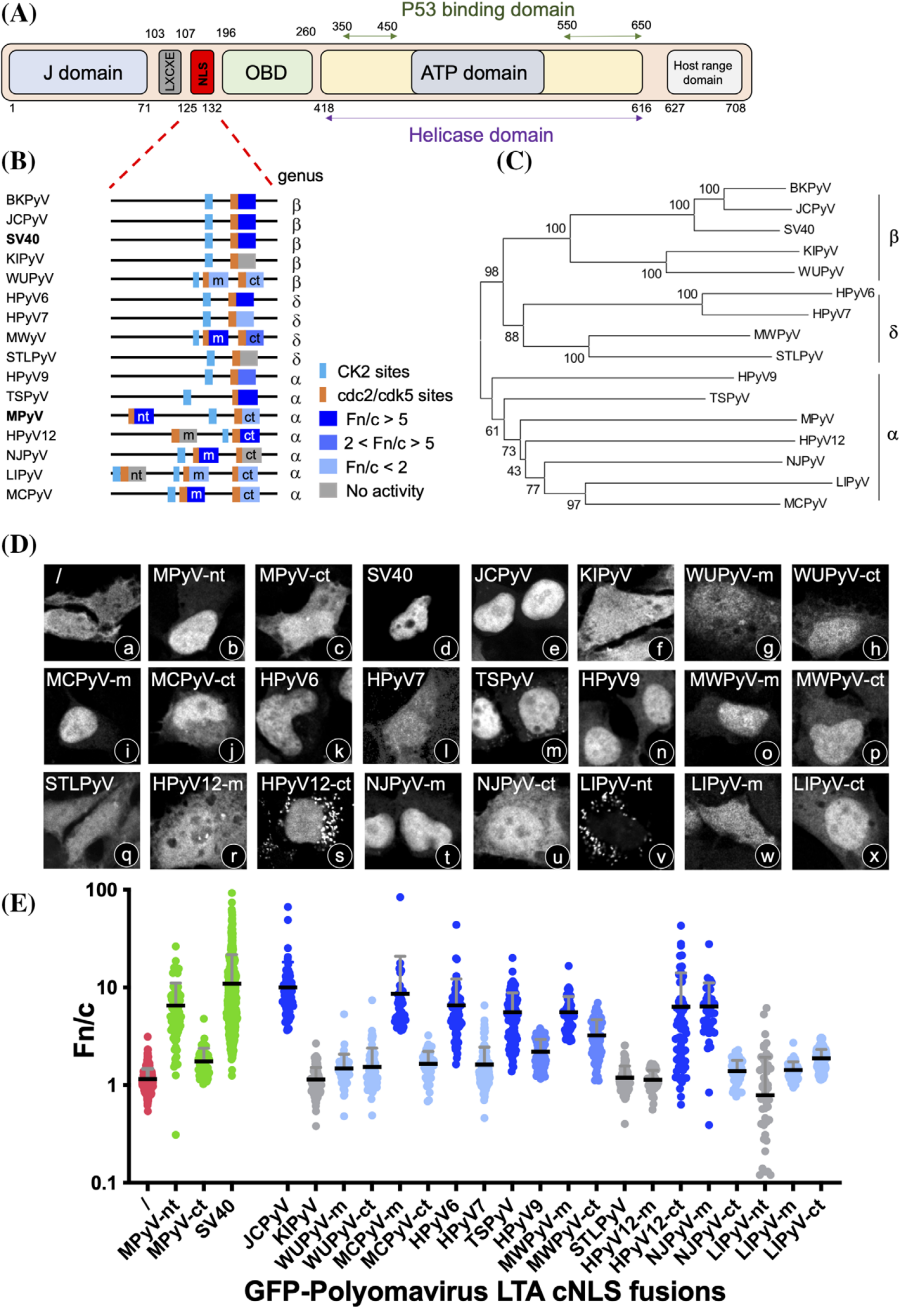
HPyV LTA cNLSs are extremely heterogeneous. (A) A schematic representation of HPyV LTA domains, using the SV40 LTA aa positions as a reference. (B) Distribution of putative cNLSs identified in the region between the LXCXE motif and the OBD domain. The protein sequence is represented as a black line. cNLSs are shown as rectangles colored according to the mean Fn/c relative to each GFP‐NLS fusion calculated in panels DE. Gray boxes indicate no significant difference compared to GFP alone, as assessed by the student's *t*‐test with Welch's correction, whereas colored boxes indicate significant differences in Fn/c: dark blue, Fn/c > 5; medium blue, 2 < Fn/c < 5; light blue, Fn/c < 2. CK2 phosphorylation sites are represented as blue vertical lines. Cdc2/cdk5 phosphorylation sites are shown as orange vertical lines. (C) Phylogenetic analysis of LTAs from all HPyVs, performed using Clustal W. (D) Cell micrographs of HEK293A cells transfected for 48 h with plasmids encoding the indicated GFP‐fusion proteins, before being processed for immunofluorescence (IF) and quantitative CLSM. (E) Cell micrographs, such as those in (D), were used to calculate the Fn/c relative to single cells expressing the indicated GFP‐fusion proteins. Each filled circle corresponds to quantification of a single cell. Fn/c values relative to GFP alone are in red, those relative to GFP fused to positive control LTA cNLS sequences (MPyV‐nt, MPyV‐ct, and SV40) are in green, and those from tested PyV LTA GFP‐fusions are colored according to their mean Fn/c as in (B). CLSM, confocal laser scanning microscopy; cNLS, classical nuclear localization signal; LTA, large tumor antigen; NLS, nuclear localization signal; OBD, origin binding domain.

SV40 LTA is the prototypical and best described cNLS. To identify putative cNLSs within other HPyV LTAs, we bioinformatically analyzed the aa sequences of LTAs from all HPyVs, using sequences from SV40 and MPyV LTA as a reference. Sequences were aligned with Clustal W and putative cNLSs were identified with cNLS mapper and by visual inspection of basic aa clusters. SV40 LTA contains a single cNLS located between the LXCXE motif and the OBD (126‐P**KKKRK**V‐132), whereas MPyV LTA contains two cNLSs, one located in a similar position to SV40 LTA (279‐PP**KK**A**R**ED‐286) and one located upstream (188‐PVS**RKR**P**R**PA‐197) (Figure 2A–C). Our analysis identified at least one putative cNLS in all HPyV LTAs (Figure 2B, Figure S1A,B, Table S1). Importantly, most cNLSs clustered downstream of the LXCXE motif, similar to the cNLSs originally described for SV40 and MPyV LTAs. Surprisingly, more than a single putative cNLS is present in several HPyVs. Such cNLSs could be assigned to either a C‐terminal position (NLSct, corresponding to the position of SV40 LTA NLS), an N‐terminal position (NLSnt, corresponding to the upstream MPyV LTA NLS), or a position in the middle (NLSm; see Table S1, Figure 2B, and Figure S1A). To functionally characterize the newly identified putative cNLSs, we transfected HEK293A cells with recombinant plasmids expressing these sequences fused to GFP, analyzed their subcellular localization by confocal laser scanning microscopy (CLSM; Figure 2D), and quantitatively analyzed the nuclear accumulation of the expressed GFP fusions at the single cell level (Figure 2E). Plasmids mediating expression of either GFP alone or GFP fused to the NLSs from SV40 and MPyV LTAs were also transfected as negative and positive controls for nuclear accumulation, respectively. As expected, GFP was evenly distributed between the nucleus and cytoplasm (Figure 2Da) with a Fn/c of 1.2 ± 0.3, while GFP‐SV40 LTA NLS strongly accumulated in the cell nucleus (Figure 2Dd) with a Fn/c of 11.0 ± 0.8. Lower levels of nuclear accumulation were calculated for GFP‐MPyV LTA NLSnt (Figure 2Db) with a Fn/c of 6.5 ± 4.6, and GFP‐MPyV LTA NLSct (Figure 2Dc) with a Fn/c of 1.7 ± 0.6. Overall, NLS activity was extremely heterogeneous, and the putative cNLSs identified here could be classified according to their nuclear targeting activity when fused to GFP. John Cunningham PyV (JCPyV) LTA NLS (Figure 2De), MCPyV LTA NLSm (Figure 2Di), HPyV6 LTA NLS (Figure 2Dk), Trichodysplasia spinulosa PyV (TSPyV) LTA NLS (Figure 2Dm), Malawi PyV (MWPyV) LTA NLSm (Figure 2Do), HPyV12 LTA NLSct (Figure 2Ds), and New Jersey PyV (NJPyV) LTA NLSm (Figure 2Dt) all mediated strong nuclear accumulation, with a Fn/c > 5 (Figure 2E, *dark blue circles*). HPyV9 LTA NLS (Figure 2Dn) and MWPyV LTA NLSct (Figure 2Dp) conferred intermediate levels of nuclear accumulation (2 < Fn/c < 5, Figure 2E, *medium blue circles*). On the other hand, the activity of NJPyV LTA NLSct (Figure 2Du), Lyon IARC PyV (LIPyV) LTA NLSm (Figure 2Dw), Washington University PyV (WUPyV) LTA NLSm (Figure 2Dg), WUPyV LTA NLSct (Figure 2Dh), HPyV7 LTA NLS (Figure 2Dl), MCPyV LTA NLSct (Figure 2Dj), and LIPyV LTA NLSct (Figure 2Dx) were rather weak (Fn/c < 2, Figure 2E, *light blue circles*). Only LIPyV LTA NLSnt (Figure 2Dv), HPyV12 LTA NLSm (Figure 2Dr), KIPyV LTA NLS (Figure 2Df), and STLPyV LTA NLS (Figure 2Dq) were completely devoid of nuclear targeting ability and did not confer a statistically significant increase of nuclear accumulation compared to GFP (Figure 2E, *gray circles*). A summary of quantitative data is shown in Table S2. At least one functional cNLS was identified for each HPyV LTA, except for KIPyV and STLPyV, where no functional monopartite cNLSs were found. Intriguingly, WUPyV, MWPyV, LIPyV, and MCPyV LTAs possess two closely located functional monopartite cNLSs (see also Figure 2B), whereas HPyV7 LTA possesses only one extremely weak cNLS (Fn/c = 1.6 ± 0.8). Overall, there was a weak, positive correlation between cNLS activity and cNLS mapper scores (Figure S2).

### 2.2 HPyV LTAs are transported to the nucleus by the IMPα/β heterodimer

**FIGURE 3:**
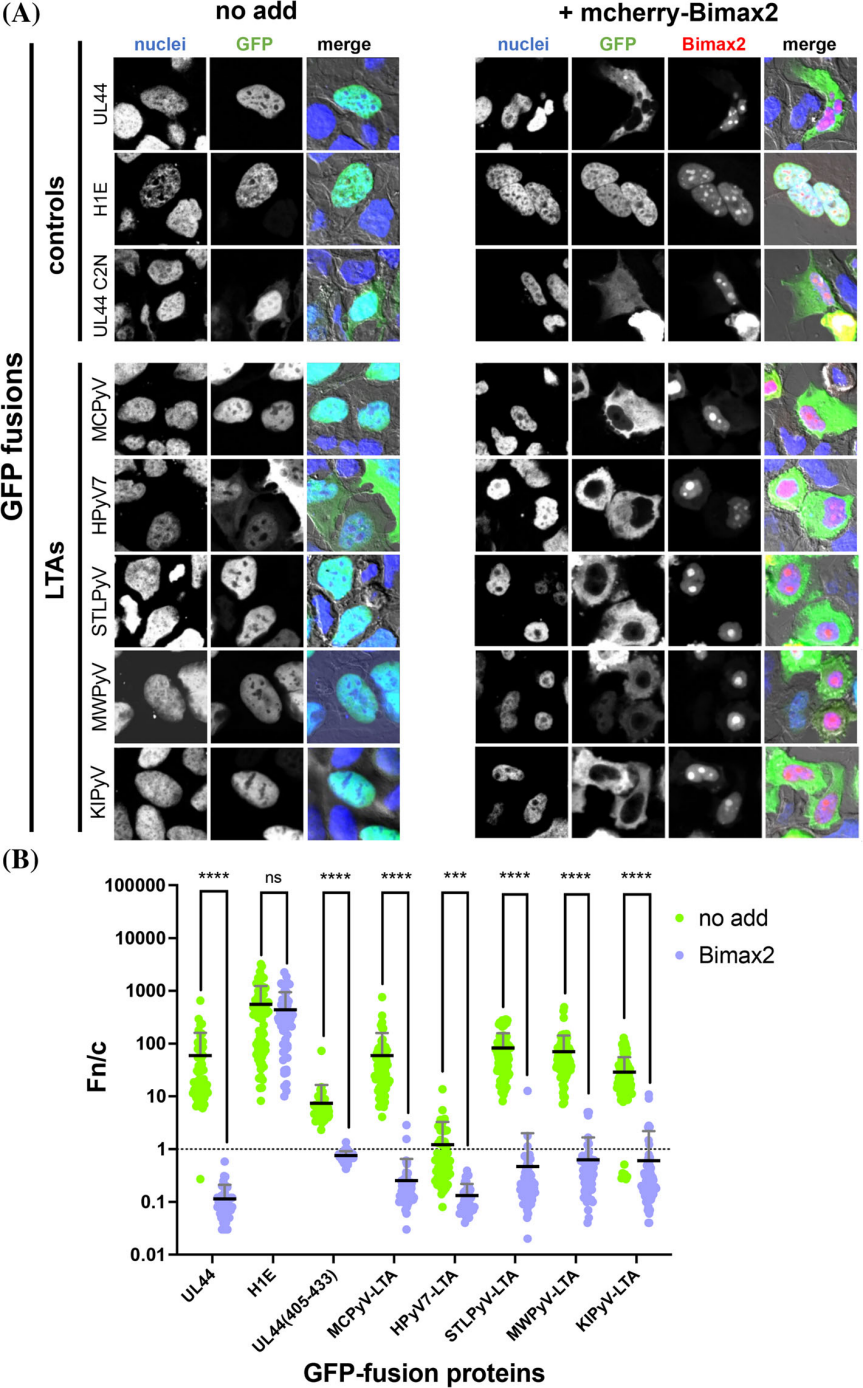
Nuclear accumulation of HPyV LTAs is dependent on the IMP⍺/β heterodimer. (A) HEK293A cells were transfected with plasmids encoding the indicated GFP‐fusion proteins in the absence (*left* panels, no add) or presence (*right* panels, + mcherry‐Bimax2) of a plasmid encoding for mcherry‐Bimax2. At 48 h p.t., cells were processed for IF and subcellular localization of GFP‐fusion proteins was quantitatively analyzed by CLSM. The DRAQ5 (nuclei), GFP (GFP), and mcherry (Bimax2) channels are shown, along with a merged image containing the bright field (merge) relative to representative phenotypes. (B) Micrographs, such as those shown in (A), were quantitively analyzed to calculate the Fn/c relative to individual cells. Data shown are mean ± standard error of the mean (SEM) relative to each GFP‐fusion, including the results of the student's *t*‐test for significance between expression in the absence (green circles) or presence (purple circles) of mcherry‐Bimax2. ****p < 0.0005; ***p < 0.005. CLSM, confocal laser scanning microscopy; LTA, large tumor antigen.

Given their role in viral genome expression and replication within the cell nucleus, the absence of a functional monopartite cNLS on KIPyV and STLPyV LTAs suggested that their nuclear transport might be dependent on non‐classical NLSs via an IMPα/β‐independent pathway. To address this, we expressed full‐length LTAs from KIPyV and STLPyV in the presence or absence of mcherry‐Bimax2, a well‐known inhibitor of the IMPα/β‐mediated nuclear transport pathway (Kosugi et al., 2008), and analyzed their subcellular localization by CLSM (Figure 3). We also expressed LTAs from other HPyVs which are likely imported by the IMPα/β heterodimer similar to SV40 LTA. These include MCPyV LTA, which possesses two cNLSs (the previously described NLSm, 274‐PFSR**KRK**FGGS‐284, and the newly identified NLSct, 299‐PP**K**P**KK**N**R**E‐307), MWPyV LTA, which possesses two closely located cNLSs (NLSm and NLSct), as well as HPyV7 LTA, which bears an extremely weak cNLS. GFP‐UL44, the human cytomegalovirus (HCMV) DNA polymerase processivity factor, a c. 90 kDa fusion protein which translocates into the nucleus via the IMPα/β heterodimer, GFP‐UL44‐C2N, a c. 30 kDa monomeric protein containing residues 405–433 of UL44 (Alvisi et al., 2005), and GFP‐H1E, a 45 kDa fusion protein endowed with dsDNA binding ability which is imported into the nucleus by multiple import pathways (Jakel et al., 1999), were also expressed as additional controls. As expected, in the absence of mcherry‐Bimax2, such positive controls accumulated in the cell nucleus (Figure 3A, *left panels*, Figure 3B, *green circles*, Figure S3). Interestingly, while MCPyV, MWPyV, STLPyV, and KIPyV LTA‐GFP fusions strongly localized to the cell nucleus (Fn/c > 5) in almost every transfected cell, subcellular localization of HPyV7 LTA‐GFP was extremely heterogeneous, accumulating in the nucleus in only 30% of cells (Fn/c = 1.2 ± 2.1, Figure 3A, *left panels*, Figure 3B, *green circles*, Figure S3, Table S3). As expected, expression of mcherry‐Bimax2 resulted in complete relocalization of GFP‐UL44 to the cytoplasm, consistent with its high molecular weight and dependence on IMPα/β for nuclear transport (Figure 3A, *right panels*, Figure 3B, *purple circles*, Figure S3). On the other hand, GFP‐UL44‐C2N exhibited a diffuse pattern throughout cells expressing mcherry‐Bimax2, indicative of its ability to passively diffuse through the NPC, while GFP‐H1E subcellular localization was unaffected by overexpression of mcherry‐Bimax2, consistent with its ability to strongly bind dsDNA and be transported into the nucleus by multiple transport pathways. Importantly, the presence of mcherry‐Bimax2 strongly inhibited nuclear import of all HPyV LTAs tested (Figure 3A, *right panels*), resulting in cytoplasmic retention (Fn/c < 1, Figure 3B, *purple circles*) in almost 100% of cells (Figure S3). A summary of quantitative data is shown in Table S3. Therefore, all tested HPyV LTAs, including those from KIPyV and STLPyV, are translocated into the nucleus by the IMPα/β heterodimer. Further, HPyV7 LTA, which possesses an extremely weak monopartite cNLS, is efficiently imported into the nucleus, yet only in a fraction of cells.

### 2.3 Several HPyV LTAs contain bipartite cNLSs

**FIGURE 4:**
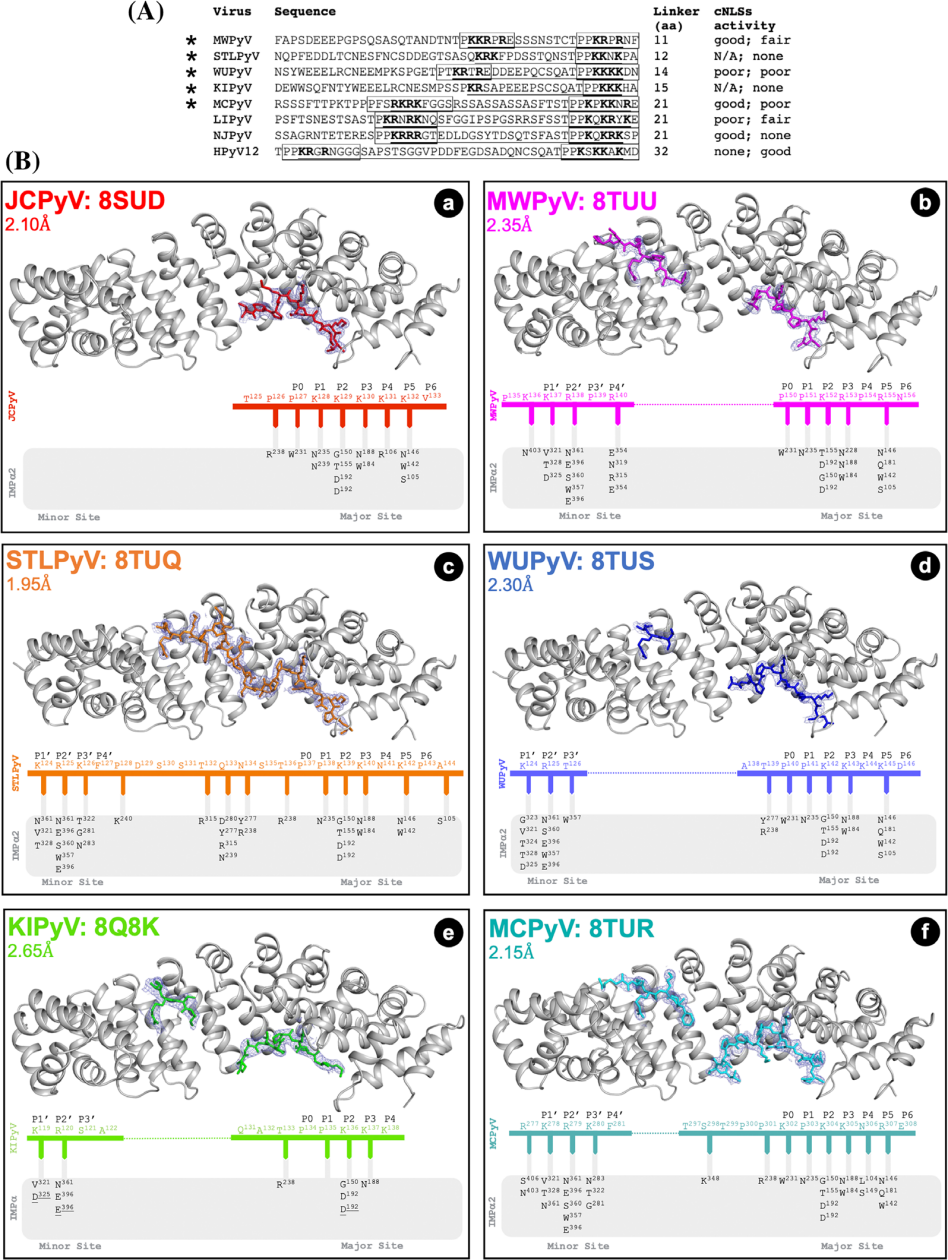
Several HPyV LTAs contain bipartite cNLSs. (A) Putative bipartite cNLSs are present in HPyV LTAs. The sequences corresponding to the NLS regions of the LTAs from the indicated HPyVs bound to IMP⍺ are shown using the single letter aa code. Putative monopartite cNLS are boxed, and their activity when fused to GFP is indicated (none: no statistical difference from GFP alone; poor: 1 < Fn/c < 2; fair: 2 < Fn/c < 5; good: Fn/c > 5). Putative bipartite cNLSs are underlined, and the distance between the two basic stretches of aas forming them is indicated. * = bipartite NLSs that have been characterized in this study. (B) Crystal structures of the indicated HPyV LTA NLSs bound to mIMP⍺2ΔIBB, including their PDB ID. Gray cartoon represents IMP⍺2ΔIBB and colored sticks represent HPyV LTA cNLSs. A schematic representation of the binding interactions is shown below each structure, detailing hydrogen bonds and salt bridges (underlined). In panels (b, d–f), the dotted line represents bipartite cNLS linker residues that were not modeled in the crystal structures due to missing electron density from inherent flexibility of this region. PDBePISA was used for all binding interaction calculations. a: JCPyV LTA NLS (red, PDB ID: 8SUD); b: MWPyV LTA NLSbip (magenta, PDB ID: 8TUU); c: STLPyV LTA NLSbip (orange, PDB ID: 8TUQ); d: WUPyV LTA NLSbip, (blue, PDB ID: 8TUS); e: KIPyV LTA NLSbip (green, PDB ID: 8Q8K), only chains B and D from this structure were included for comparison in the figure; f: MCPyV LTA NLSbip (cyan, PDB ID: 8TUR). The peptide sequences used for crystallization are listed in Table S9. The 2FO‐FC electron density surrounding each NLS peptide is shown in light blue isomesh contoured to 1.0 sigma within 1.5 Å of selected atoms, with the exception of KIPyV LTA NLSbip where the peptide is contoured to 1.0 sigma within 1.8 Å of selected atoms. cNLS, classical nuclear localization signal; LTA, large tumor antigen; NLS, nuclear localization signal.

The fact that STLPyV and KIPyV LTAs localize into the nucleus in an IMPα/β‐dependent fashion despite lacking a functional monopartite cNLS prompted us to further analyze their sequences. Interestingly, we noticed additional basic aas present upstream of their non‐functional monopartite cNLSs (Figure S1A,B). A closer inspection of the cNLS clusters of all HPyV LTAs revealed that in several cases, multiple monopartite cNLSs are closely located, potentially forming bipartite cNLSs (Figure S1C). The linkers between the two stretches of basic aas range from 11 aas in MWPyV to 32 aas in HPyV12 (Figure 4A). We therefore reasoned that nuclear import of several HPyV LTAs, including those from STLPyV and KIPyV, could be mediated by bipartite cNLSs. We addressed this possibility by x‐ray crystallography, and solved the structures of putative bipartite cNLS peptides of MWPyV, STLPyV, WUPyV, KIPyV, and MCPyV LTAs bound to IMPα2ΔIBB. We also included JCPyV and HPyV7 LTA cNLSs as controls for strong and weak monopartite cNLSs, respectively. All structures of IMPα2ΔIBB and NLS peptides were resolved between 1.95 and 2.65 Å, except for HPyV7 LTA NLS for which no complex was structurally characterized (Figure 4B). Crystallization conditions as well as collection and refinement statistics are shown in Tables S4 and S5. As expected, JCPyV LTA cNLS bound as a monopartite cNLS at the major site of IMPα2ΔIBB, with no electron density for the peptide observed at the minor site (Figure 4Ba). The thermodynamically dominant P2 pocket of IMPα2ΔIBB was occupied by JCPyV LTA cNLS K129, making interactions with IMPα2ΔIBB through hydrogen bonds (G150, T155, D192) and a salt bridge (D192). Strikingly, all other tested cNLS peptides bound as bipartite cNLSs (NLSbip), occupying both the minor and major binding sites of IMPα2ΔIBB with their upstream and downstream basic clusters, respectively (Figure 4Bb–f). Interestingly, crystallization of the KIPyV LTA:IMPα2ΔIBB complex resulted in an open dimer conformation between the two IMPα molecules, bridged by two disulfide bonds at Cys133/223 in both chains A and B. The concave binding region remained accessible on each IMPα and the KIPyV LTA NLS bound as a bipartite cNLS similarly on chain A and B. We don't believe this to be related to the ability of KIPyV to bind IMPα differently to the other LTAs, rather a result of the crystallization condition. In the STLPyV LTA NLSbip structure, electron density for the entire cNLS including the linker region was clearly resolved (Figure 4Bc). For all bipartite cNLSs, the IMPα2ΔIBB P2 major site pocket is occupied by a K residue, forming hydrogen bonds with IMPα2ΔIBB residues and a salt bridge with D192, whereas the P2’ minor site pocket is occupied by an R residue, forming hydrogen bonds with IMPα2ΔIBB residues and a salt bridge with E396. As expected, the major site P3 and P5 pockets are occupied by either K or R residues, establishing hydrogen bonds with IMPα2ΔIBB residues, except in the case of KIPyV LTA NLS where P5 was not modeled (Figure 4Be). The P4 position is occupied by either P, N, or K residues, which, only in the case of N306 from MCPyV LTA cNLS and K131 from JCPyV LTA cNLS, interact via hydrogen bonds with IMPα2ΔIBB residues (Figure 4Baf). Therefore, our structures show that LTAs from several HPyVs contain bipartite cNLSs, simultaneously binding to the IMPα2ΔIBB major and minor binding sites.

### 2.4 HPyV LTA cNLSs bind differently to IMPα isoforms

**FIGURE 5:**
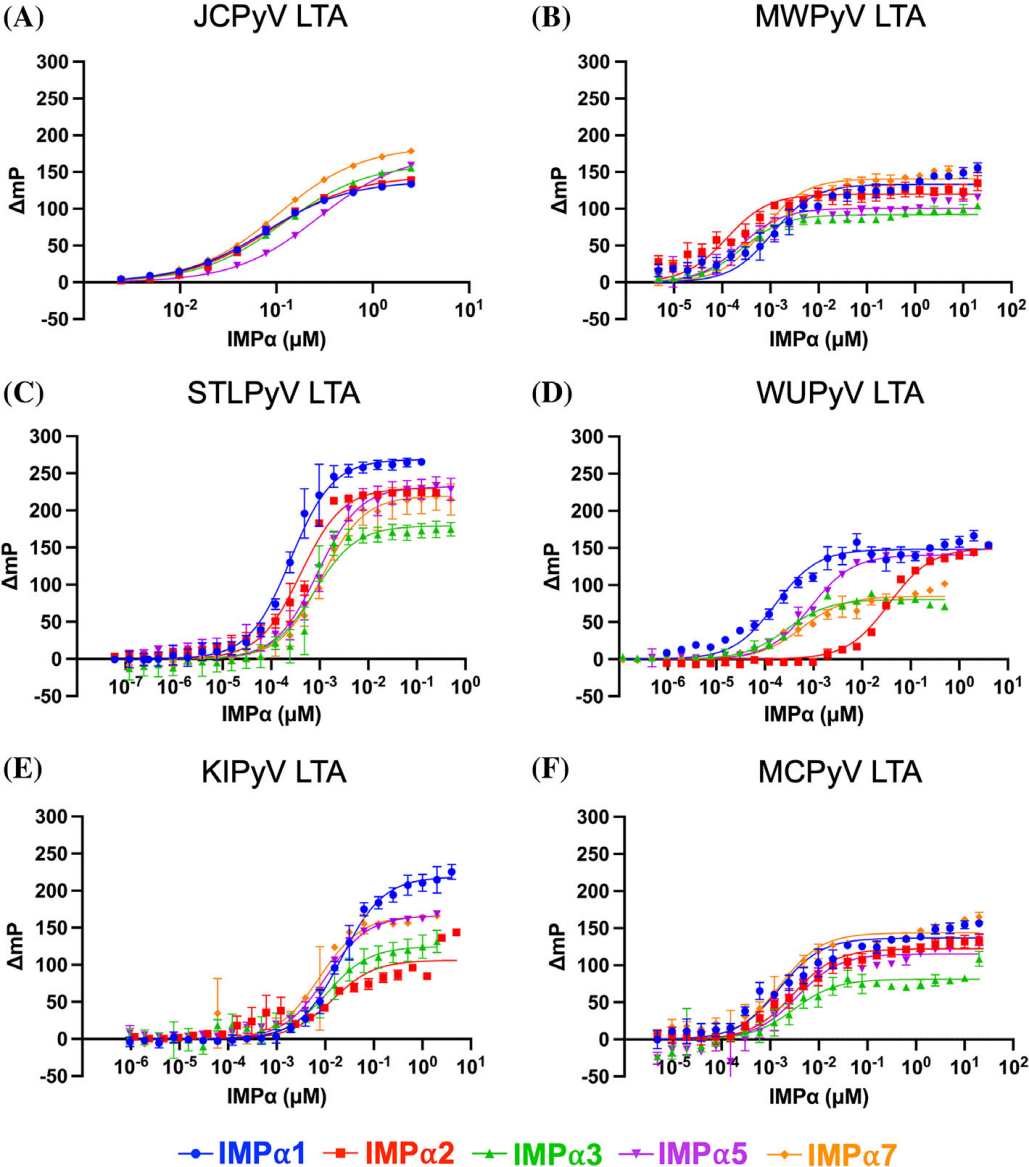
HPyV LTA cNLSs bind differently to IMP⍺ ΔIBB isoforms. FP assays were used to measure direct binding between recombinantly produced IMP⍺ΔIBB subfamily proteins and synthesized FITC‐labeled cNLS peptides. Each assay was repeated in triplicate. Error bars represent mean values ± SEM. A: JCPyV LTA NLS, FITC‐Ahx‐MFASDDENTGSQHSTPPKKKKKV‐133; B: MWPyV LTA NLSbip, FITC‐Ahx‐PKKRPRESSSNSTCTPPKRPRNF‐157; C: STLPyV LTA NLSbip, FITC‐Ahx‐KRKFPDSSTQNSTPPKKNKPA‐144; D: WUPyV LTA NLSbip, FITC‐Ahx‐KRTREDDEEPQCSQATPPKKKKD‐145; E: KIPyV LTA NLSbip, FITC‐Ahx‐KRSAPEEEPSCSQATPPKKKHA‐140; F: MCPyV LTA NLSbip, FITC‐Ahx‐PFSRKRKFGGSRSSASSASSASFTSTPPKPKKNRE‐308. cNLS, classical nuclear localization signal; LTA, large tumor antigen; NLS, nuclear localization signal.

Since IMPα isoforms are differently expressed in human tissues, cNLS composition can influence the binding affinity for IMPα isoforms (Miyamoto et al., 2016; Pumroy et al., 2015; Pumroy & Cingolani, 2015). Further, as LTA nuclear targeting is absolutely required for SV40 replication (Lanford & Butel, 1984), the ability of specific LTAs to interact with specific IMPα isoforms could play a role in determination of HPyVs tropism, similar to what has been proposed for influenza A and herpes simplex type 1 (Dohner et al., 2018; Ninpan et al., 2016). Therefore, we tested the ability of the above‐mentioned HPyV LTA bipartite cNLSs to interact with IMPαΔIBB isoforms using electrophoresis mobility shift assays (EMSAs). As controls, we included the monopartite cNLSs from JCPyV and HPyV7 LTAs. As expected, JCPyV LTA monopartite cNLS co‐migrated with all IMPαΔIBB isoforms, implying direct binding (Figure S4A). On the other hand, a very small fraction of HPyV7 LTA monopartite cNLS co‐migrated with IMPαΔIBB isoforms, indicating that the low activity of HPyV7 cNLS is due to impaired binding to IMPα (Figure S5A). All tested bipartite cNLSs clearly co‐migrated with IMPα isoforms, further confirming direct interaction (Figure S4B–F). Next, the affinities of the IMPαΔIBB:cNLS interactions were quantitatively measured by means of fluorescence polarization (FP) assays. Our results determined interaction affinities of IMPαΔIBB isoforms for each NcLS (Figure 5), except for HPyV7 LTA cNLS, whose binding to IMPαΔIBB isoforms was too weak to allow estimation of a Kd (Figure S5B). Our analysis revealed the specificity of the IMPαΔIBB:NLS interactions. The lowest Kd for JCPyV and WUPyV LTA cNLSs was measured with IMPα1ΔIBB, for KIPyV and WUPyV LTA cNLSs was with IMPα7ΔIBB, and for MWPyV LTA cNLSs was with IMPα2ΔIBB. In the case of MCPyV LTA cNLSs, the lowest Kd was measured with IMPα1ΔIBB and IMPα7ΔIBB (Table S6). Overall, bipartite cNLSs bound to IMPαΔIBB isoforms with higher affinity compared to the monopartite cNLS from JCPyV LTA. Indeed, JCPyV LTA cNLS bound to IMPαΔIBB isoforms with Kds between 76 and 270 nM, whereas bipartite cNLSs measured Kds between 0.1 and 36.1 nM (Table S6). Intriguingly, the cNLS peptides bound to IMPαΔIBB isoforms with different Bmax values. JCPyV, MWPyV, and MCPyV LTA cNLS peptides had a higher Bmax for IMPα7ΔIBB, KIPyV and STLPyV LTA cNLSs for IMPα1ΔIBB, and KIPyV LTA cNLSs for IMPα2ΔIBB (Table S6). Therefore, each HPyV LTA cNLS is endowed with a specific IMPα binding profile, and bipartite cNLSs bind to IMPαΔIBB isoforms with higher affinity than monopartite cNLSs.

### 2.5 Functional characterization of HPyV LTA bipartite cNLSs

**FIGURE 6:**
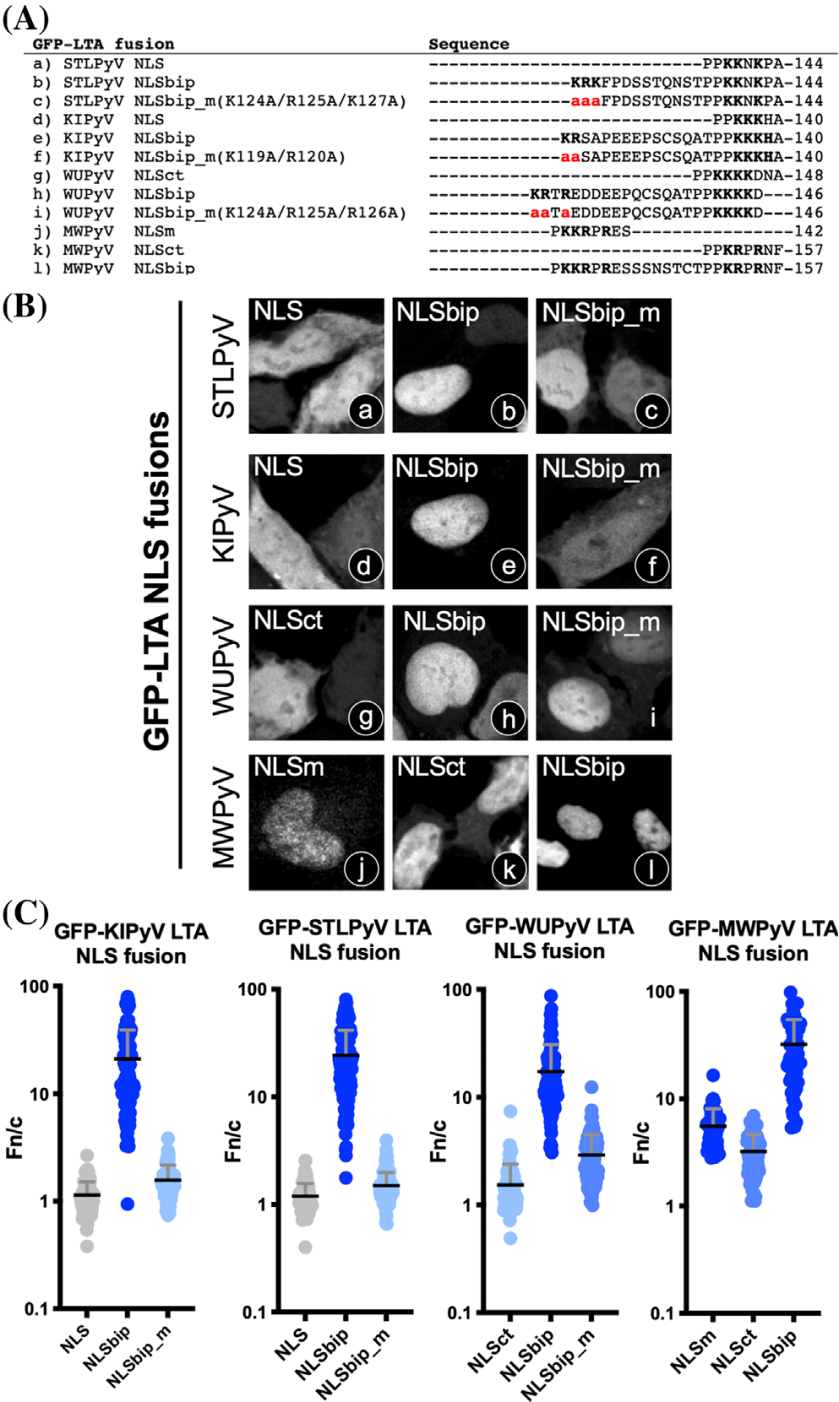
Functional characterization of bipartite cNLSs from STLPyV, KIPyV, WUPyV, and MWPyV LTAs. (A) Sequence alignment of HPyV LTA cNLS regions. The single letter aa code is used. Basic aas are in bold, and aa substitutions with respect to wt sequences are in red. HEK293A cells were transfected to express the indicated GFP fusion proteins, before being processed for CLSM (B) and quantitative analysis of the Fn/c ratios relative to individual cells (C). Data shown are mean ± SEM relative to each GFP‐fusion. Gray circles indicate no significant difference compared to GFP alone, as assessed by the student's *t*‐test with Welch's correction, whereas colored circles indicate significant differences in terms of mean Fn/c: dark blue, Fn/c > 5; medium blue, 2 < Fn/c < 5; light blue, Fn/c < 2. CLSM, confocal laser scanning microscopy; cNLS, classical nuclear localization signal; LTA, large tumor antigen.

Our structural and biochemical analyses revealed that KIPyV, STLPyV, WUPyV, and MWPyV LTAs possess bipartite cNLSs. We therefore decided to investigate their functionality in terms of nuclear transport compared to their monopartite counterparts (Figure 6A). To this end, cells transiently expressing several cNLS derivatives fused to GFP were analyzed by CLSM (Figure 6B), followed by quantitative analysis of the levels of nuclear accumulation (Figure 6C). For STLPyV and KIPyV LTAs, which are devoid of a functional monopartite cNLS, we compared nuclear targeting activity of the bipartite cNLSs to that of the putative monopartite cNLSs and to that of the bipartite cNLSs whereby the basic aas accommodated at the minor site were substituted with A (NLSbip_m) (Figure 6A). As expected, neither STLPyV LTA cNLS (Figure 6Ba) nor KIPyV LTA cNLS (Figure 6Bd) targeted GFP to the nucleus, in stark contrast to both STLPyV LTA NLSbip (Figure 6Bb) and KIPyV LTA NLSbip (Figure 6Be), which conferred strong nuclear localization. Intriguingly, both GFP‐STLPyV LTA NLSbip_m, bearing the K124A/R125A/K127A substitutions (Figure 6Bc), and GFP‐KIPyV LTA NLSbip_m, bearing the K119A/R120A substitutions (Figure 6Bf), were considerably less nuclear (Figure 6C). Similar results were obtained for GFP‐WUPyV LTA NLSbip (Figure 6Bh), which localized to the nucleus to higher levels (Figure 6C) than GFP‐WUPyV LTA NLSct (Figure 6Bg) and the derivative GFP‐WUPyV LTA NLSbip_m, bearing the K124A/R125A/R126A substitutions (Figure 6Bi). Similarly, in the case of MWPyV LTA, the fusion protein between GFP and NLSbip (Figure 6Bl) was significantly more nuclear than both GFP‐MWPyV LTA NLSm (Figure 6Bj) and GFP‐MWPyV LTA NLSct (Figure 6Bk). Quantitative data are summarized in Figure 6C and strongly suggest that binding of basic residues at the IMPα minor binding site plays a crucial role in cNLS activity of KIPyV, STLPyV, WUPyV, and MWPyV LTAs.

### 2.6 cNLSs located between the LXCXE motif and OBD are essential for nuclear targeting of HPyV LTAs

**FIGURE 7:**
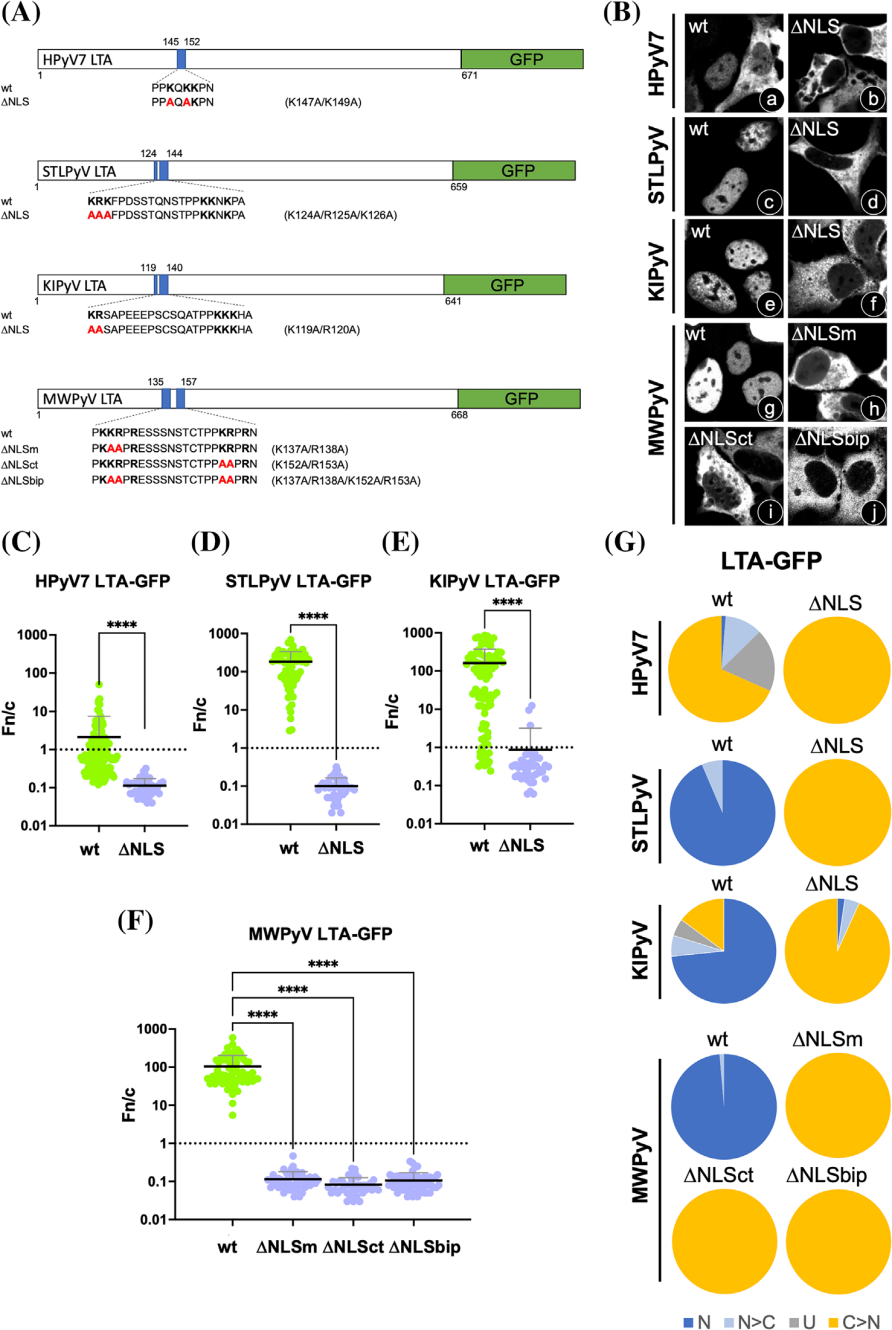
Identification of residues essential for nuclear transport of HPyV LTAs. The subcellular localization of the indicated HPyV LTAs fused to the N‐terminus of GFP (A) was analyzed in transfected HEK293A cells. (B) At 48 h p.t., cells were processed for IF and the subcellular localization of GFP‐fusion proteins was analyzed by quantitative CLSM. Cell micrographs, such as those shown in (B), were used to quantify the Fn/c relative to single cells expressing the HPyV7 (C), STLPyV (D), KIPyV (E), and MWPyV (F) LTA‐GFP fusion proteins and NLS defective derivatives. Data shown are the mean and standard deviation of the mean (SD) relative to single cells, with indicated significance scores from the student's *t*‐test between NLS defective (purple circles) and wt (green circles) proteins. ****p < 0.0005. (G) The percentage of cells relative to each indicated fusion protein displaying the indicated subcellular localizations is shown. N: nuclear, Fn/c ≥ 10; N > C: nuclear more than cytosolic, 2 ≤ Fn/c < 10; U: ubiquitous, 1 ≤ Fn/c < 2; C > N: more cytosolic than nuclear, Fn/c < 1. CLSM, confocal laser scanning microscopy; LTA, large tumor antigen; NLS, nuclear localization signal.

We validated the functional role of the cNLSs characterized here by testing the effect of key aa substitutions on nuclear accumulation of full‐length HPyV LTAs. To this end, we expressed LTA‐GFP fusion derivatives (Figure 7A) and analyzed their subcellular localization by CLSM (Figure 7B), before quantifying the levels of nuclear accumulation of each fusion protein at the single cell level (Figure 7C–F, Table S7). Fusion proteins tested included HPyV7 LTA and its ΔNLS substitution derivative, bearing the K147A/K149A substitutions, whereby the 144‐PP**K**Q**KK**PN‐152 cNLS sequence is replaced by 144‐PPaQa**K**PN‐152, STLPyV LTA and its ΔNLS substitution derivative, bearing the K124A/R125A/K126A substitutions at the IMPα minor binding site, whereby the 124‐**KRK**FPDSSTQNSTPP**KK**N**K**PA‐144 sequence is replaced by 124‐aaaFPDSSTQNSTPP**KK**N**K**PA‐144, KIPyV LTA and its ΔNLS substitution derivative, bearing the K119A/R120A substitutions at the IMPα minor binding site, whereby the 119‐**KR**SAPEEEPSCSQATPP**KKK**HA‐140 sequence is replaced by 119‐aaSAPEEEPSCSQATPP**KKK**HA‐140, and MWPyV LTA and its ΔNLSm, ΔNLSct, and ΔNLSbip substitution derivatives, bearing either the K137A/R138A substitutions at the IMPα minor site, the K152A/R153A substitutions at the IMPα major site, or both, whereby the 135‐P**KKR**P**R**ESSSNSTCTPP**KR**P**R**NF‐157 sequence is replaced by either 135‐P**K**aaP**R**ESSSNSTCTPP**KR**P**R**NF‐157, P**KKR**P**R**ESSSNSTCTPPaaP**R**NF‐157, or 135‐P**K**aaP**R**ESSSNSTCTPPaaP**R**NF‐157, respectively (Figure 7A). All wild type (wt) proteins localized to the cell nucleus, although to differing extents. Indeed, while HPyV7 LTA‐GFP (Figure 7Ba) poorly accumulated in the cell nucleus with a Fn/c of 2.1 (Figure 7C), STLPyV (Figure 7Bc), KIPyV (Figure 7Be), and MWPyV (Figure 7Bg) LTA‐GFP all strongly accumulated in the cell nucleus, with a Fn/c > 100 (Figure 7D–F). All cNLS substitution derivatives tested were strongly impaired in nuclear targeting. HPyV7 (Figure 7Bb), STLPyV (Figure 7Bd), and KIPyV (Figure 7Bf) LTA‐GFP ΔNLS substitution derivatives were all retained in the cytoplasm, with an Fn/c < 1 (Figure 7C–E) in almost 100% of analyzed cells (Figure 7G). In the case of MWPyV LTA‐GFP, substitution of key basic residues either in the upstream (Figure 7Bh), downstream (Figure 7Bi), or both (Figure 7Bj) monopartite cNLSs completely abolished nuclear targeting (Figure 7F). This implies that despite MWPyV LTA possessing two monopartite cNLSs which are functional when fused to a heterologous protein, they only function as a bipartite cNLS in a more physiological context. These results confirm that nuclear import of HPyV7 LTA is entirely dependent on a weak monopartite cNLS, whereas nuclear import of STLPyV, KIPyV, and MWPyV LTAs depends on bipartite cNLSs located between the LXCXE motif and the OBD.

### 2.7 A hybrid NLS in MCPyV LTA

**FIGURE 8:**
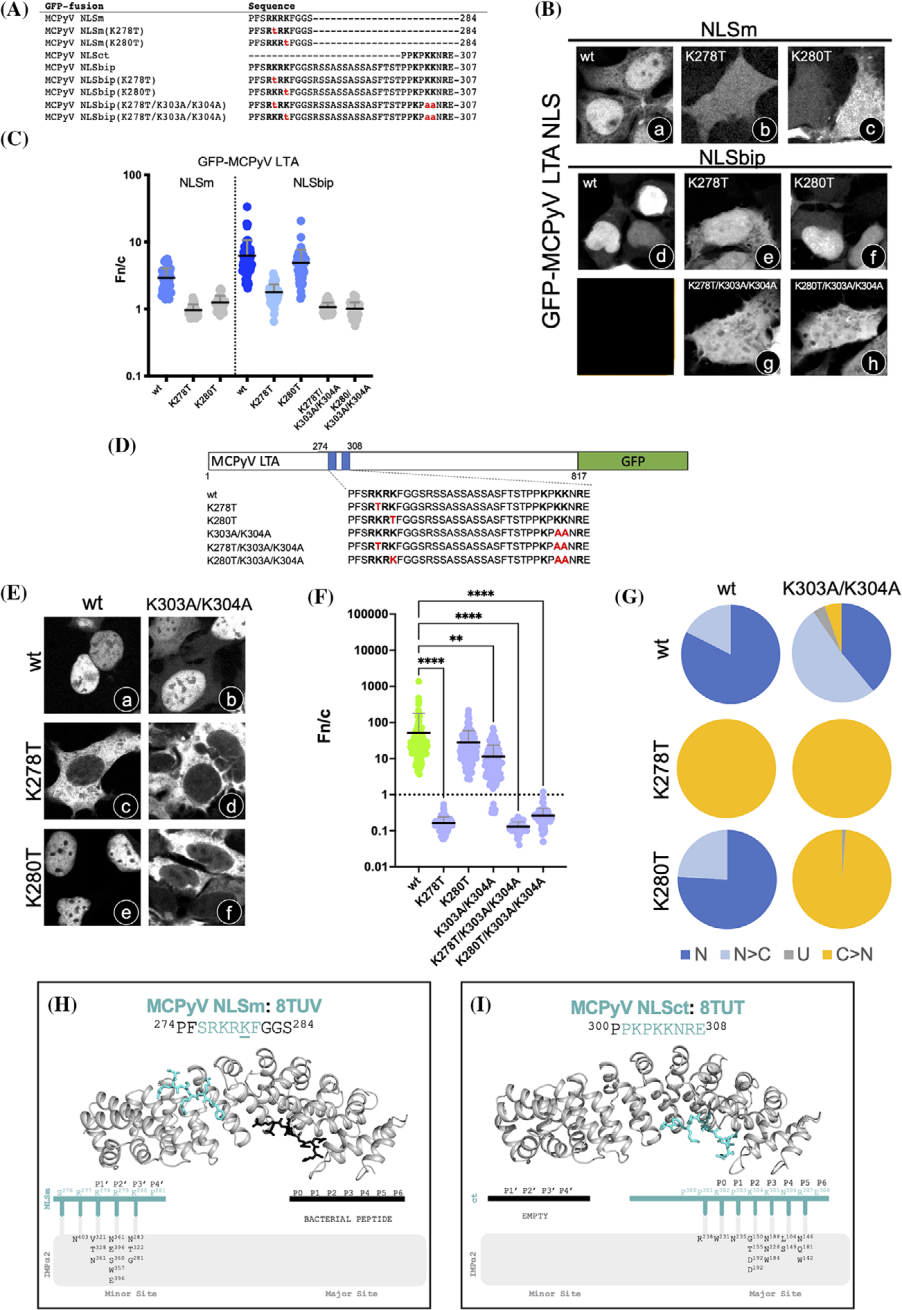
MCPyV LTA contains a hybrid NLS which can function as either a bipartite or atypical monopartite NLS. (A) HEK293A cells were transfected to express GFP‐fusions of the indicated sequences, before being processed for CLSM (B) and quantitative analysis of the Fn/c ratios relative to individual cells (C). The single letter aa code is used. Basic aas are in bold, and aa substitutions with respect to wt sequences are in red. Data shown are mean ± SEM relative to each GFP‐fusion. Gray circles indicate no significant difference compared to GFP alone, as assessed by the student's *t*‐test with Welch's correction, whereas colored circles indicate significant differences in terms of mean Fn/c: dark blue, Fn/c > 5; medium blue, 2 < Fn/c < 5; light blue, Fn/c < 2. (D) Plasmids mediating expression of the indicated full‐length MCPyV LTA‐GFP fusion proteins were used to transfect HEK293A cells. (E) At 48 h p.t., cells were processed for IF and the subcellular localization of GFP‐fusion proteins was analyzed by quantitative CLSM. (F) Cell micrographs, such as those shown in (E), were used to quantify the Fn/c ratio relative to single cells expressing the MCPyV LTA‐GFP fusion protein derivatives. Data shown are mean and SD relative to single cells, with indicated significance scores from the student's *t*‐test between cNLS defective (purple circles) and wt (green circles) proteins. ****p < 0.0005; **p < 0.01. (G) The percentage of cells relative to each indicated fusion protein displaying the indicated subcellular localizations is shown. N: nuclear, Fn/c ≥ 10; N > C: more nuclear than cytosolic, 2 ≤ Fn/c < 10; U: ubiquitous, 1 ≤ Fn/c < 2; C > N: more cytosolic than nuclear, Fn/c < 1. (H) The crystal structure of IMPa2ΔIBB with MCPyV LTA NLSm bound at the minor site is shown (PDB: 8TUV). A bacterial peptide occupies the major site. (I) The crystal structure of IMPa2ΔIBB with MCPyV LTA NLSct bound at the major site is shown (PDB: 8TUT). Gray cartoon represents IMP⍺2ΔIBB and colored sticks represent MCPyV LTA cNLSs. A schematic representation of the binding interactions is shown below each structure, detailing hydrogen bonds and salt bridges (underlined). PDBePISA was used for all binding interaction calculations. CLSM, confocal laser scanning microscopy; cNLS, classical nuclear localization signal; LTA, large tumor antigen; NLS, nuclear localization signal.

For MCPyV LTA, nuclear import so far has been believed to rely on a monopartite cNLS (NLSm: 274‐PFS**RKRK**‐280). However, our structural and biochemical data suggest that MCPyV LTA NLSm could also function as a bipartite cNLS, together with the monopartite cNLS located 20 aas downstream (NLSct: 300‐PP**K**P**KK**N**R**E‐308, Figure 2B, Figure 4A, Figure S1C). Indeed, previous studies demonstrated that in the context of full‐length MCPyV LTA, the K278T substitution completely abrogates nuclear import, whereas the K280T substitution has no effect (Nakamura et al., 2010). To shed light on this issue, we compared the effect of such substitutions on nuclear import of the GFP‐MCPyV LTA NLSm (274‐PFS**RKRK**‐280) and NLSbip (274‐PFS**RKRK**FGGSRSSASSASSASFTSTPPKP**KK**N**R**E‐308) fusion proteins. To this end, several expression plasmids (Figure 8A) were used to transfect HEK293A cells, before CLSM imaging (Figure 8B) and quantitative analysis of nuclear localization levels (Figure 8C). As expected, GFP‐MCPyV LTA NLSbip (Figure 8 Bd) localized to the nucleus to higher levels (Figure 8C) with respect to GFP‐MCPyV LTA NLSm (Figure 8Ba). Substitution K278T completely abolished targeting of both GFP‐MCPyV LTA NLSm (Figure 8Bb) and NLSbip (Figure 8Be). However, substitution K280T abolished nuclear targeting of GFP‐MCPyV LTA NLSm (Figure 8Bc) but not NLSbip (Figure 8Bf). Further, substitutions K304A/K305A, involving NLSct residues interacting with the IMPα major site P2 and P3 pockets, abolished nuclear targeting of the GFP‐MCPyV LTA NLSbip K280T protein (Figure 8Bh). These data are consistent with the idea that residues in the P1′ position (K278) of a bipartite cNLS contribute much more to IMPα binding compared to residues in the P3′ position (K280). Similar results were obtained by biochemical assays, whereby the ability of FITC‐labeled MCPyV LTA NLS peptides (Figure S6A) to bind IMPα2ΔIBB was assessed by EMSAs (Figure S6B) and FP assays (Figure S6C). Consistent with the subcellular localization data, MCPyV LTA NLSbip bound to IMPα2ΔIBB with higher affinity than MCPyV LTA NLSm. Furthermore, the K280T substitution completely abrogated IMPα2ΔIBB binding to MCPyV LTA NLSm but not NLSbip (Figure S6B,C). Taken together, our data suggest that MCPyV LTA possesses a bipartite NLS, similar to that reported above for STLPyV, KIPyV, and MWPyV LTAs.

The previous demonstration that MCPyV LTA (1–280) is still capable of accumulating into the cell nucleus (Nakamura et al., 2010) strongly suggests that in the absence of downstream residues, MCPyV LTA NLSm can function as a monopartite cNLS. We addressed this issue by combining CLSM and crystallographic approaches. First, we analyzed the effect of aa substitutions of key basic residues in MCPyV LTA NLSm (K278T/K280T) and NLSct (K303A/K304A) on nuclear accumulation of full‐length MCPyV LTA‐GFP fusion proteins (Figure 8D) after transient expression in HEK293A cells. As mentioned above, wt MCPyV LTA‐GFP strongly accumulated in the cell nucleus (Figure 8Ea), with an average Fn/c of c. 50 (Figure 8F) and a Fn/c > 2 in 100% of analyzed cells (Figure 8G). Importantly, the K303A/K304A substitutions (Figure 8Eb) reduced, but did not abolish, nuclear import, with an average Fn/c of c. 12 (Figure 8F) and a Fn/c > 2 in >90% of cells analyzed (Figure 8G). These findings confirm that in the absence of downstream basic residues interacting with the IMPα major binding site, MCPyV LTA NLSm can mediate nuclear import of the full‐length protein. The K278T substitution (Figure 8Ec) completely abolished nuclear import (Fn/c = 0.16, Figure 8F), while the K280T substitution (Figure 8Ee) only marginally affected the process (Fn/c of c. 25, Figure 8F), unless combined with the K303A/K304A substitution (Fn/c c. 0.26, Figure 8Ef). These data confirm that MCPyV LTA possesses a hybrid NLS, which functions as a bipartite cNLS in the context of full‐length MCPyV LTA, and as a monopartite NLS upon inactivation of downstream residues 300‐PPKP**KK**N**R**E‐308. Indeed, LTA nuclear localization has been confirmed in several clones obtained from Merkel Cell Carcinoma (MCC) samples, whereby the viral genome integrates in host cell chromosomes, disrupting the LTA coding sequence upstream of residues 300‐PPKP**KK**N**R**E‐308 (Houben et al., 2015).

### 2.8 MCPyV LTA NLSm is an atypical cNLS binding to the IMPαΔIBB minor binding site

In agreement with that previously reported (Nakamura et al., 2010), MCPyV LTA NLSm drives GFP to the cell nucleus (Figure 2Di, Figure 8Ba) and binds IMPα2ΔIBB (Figure S6C). However, its sequence (274‐PFS**RKRK**‐280) does not match the monopartite cNLS consensus (**K**‐**K**/**R**‐X‐**K**/**R**), therefore it could represent an atypical NLS interacting with the IMPα major binding site in a non‐canonical fashion. We addressed this issue by solving the crystal structures of IMPα2ΔIBB in complex with FITC‐labeled peptides encompassing MCPyV LTA NLSm (274‐PFS**RKRK**FGGS‐284) and NLSct (300‐PP**K**P**KK**N**R**E‐308), to resolutions of 2.30 and 2.55 Å, respectively. Our results strikingly revealed that when incubated with IMPα2ΔIBB, MCPyV LTA NLSm could be visualized exclusively within the IMPα2ΔIBB minor binding site (Figure 8H). Like in the bipartite structure (Figure 4Bf), the P1′, P2′, P3′, and P4′ sites are occupied by K278, R279, K280, and F281, respectively, and showed almost identical binding patterns. Minor structural differences toward the N‐terminus of the peptide included electron density for residue S276 and loss of the K277 hydrogen bond with IMPα2ΔIBB S406 (Figure 8H). In MCPyV LTA NLSm, the IMPα2ΔIBB major site is occupied by a bacterial peptide, corresponding to a region of *E. coli* 30S ribosomal subunit S11 (7‐ARKRVRK‐13, Uniprot: A0A140N7L9) that bound during recombinant protein expression and purification. It is likely that a stronger affinity between IMPα2ΔIBB and the bipartite MCPyV LTA NLS meant that this peptide was ejected out of the major site in the bipartite crystal structure. Superposition of the MCPyV LTA NLSm structure with the minor site‐specific binder RNA helicase II/Gu⍺ NLS (PDB: 3ZIN) is shown in Figure S7. Both peptides follow the core consensus sequence K‐R‐X‐F between sites P1′–P4′. The MCPyV LTA NLSct peptide could be visualized exclusively within the IMPα2ΔIBB major binding site, with the minor site remaining empty (Figure 8I). The P0–P6 positions were occupied by the same aas bound in the bipartite MCPyV LTA NLS crystal structure (Figure 4Bf), however, only residues P300 to E308 could be built into the electron density. An additional hydrogen bond between the P3 site K305 residue and IMPα2ΔIBB N228 was also observed. We identified for the first time a hybrid bipartite NLS in MCPyV LTA, formed by two basic stretches of aas which can either simultaneously bind the IMPα minor and major binding sites as a bipartite cNLS, or, upon deletion of the genetic sequence encoding for the downstream basic residues, selectively bind to the IMPα minor binding site. The increased interaction interface observed in the bipartite cNLS structure reflects the increased binding affinity observed for this peptide in our binding assays with IMPαΔIBB isoforms.

### 2.9 Distribution of cNLSs across all known PyVs

**FIGURE 9:**
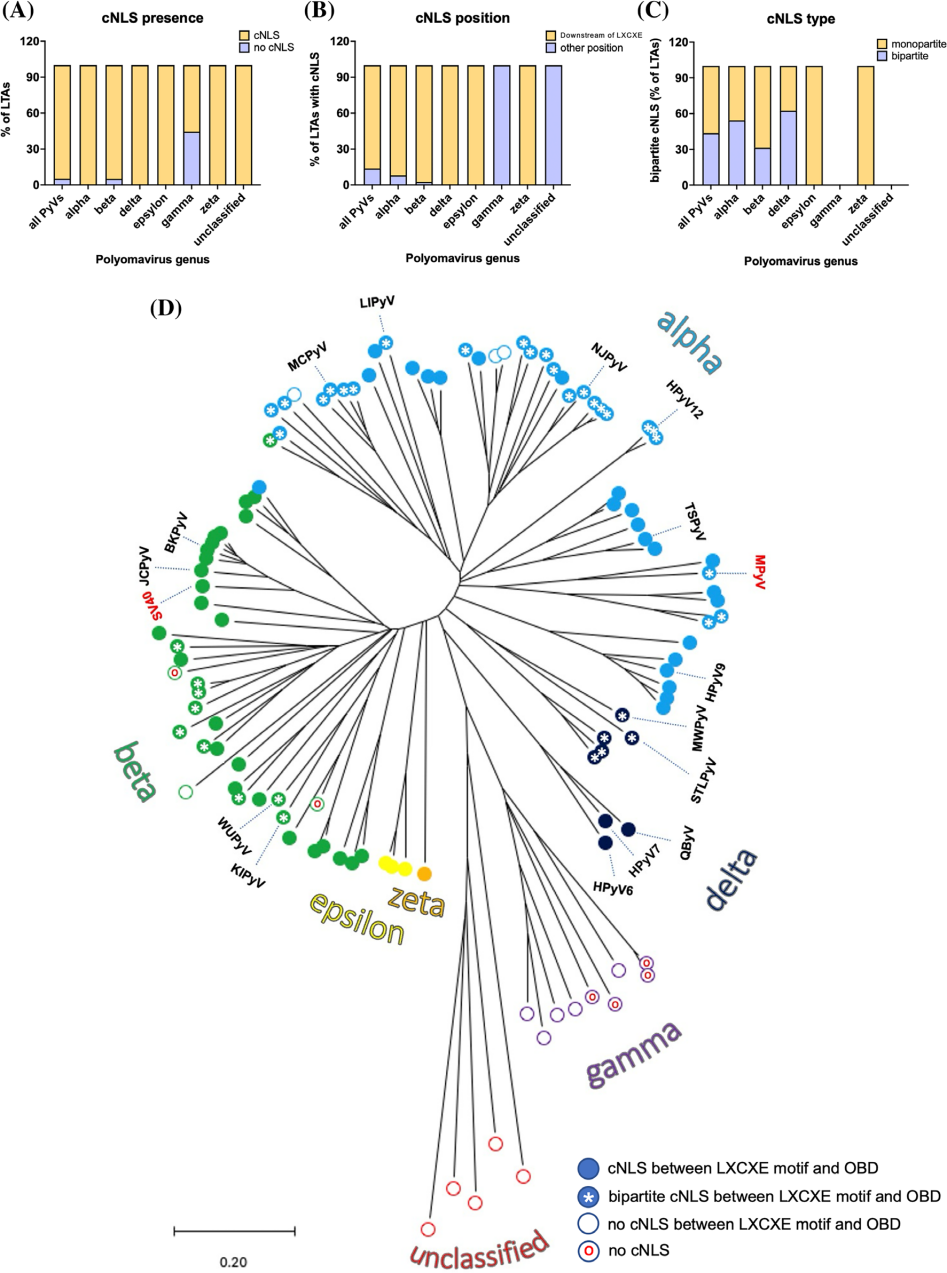
Distribution of cNLSs on all known PyV LTAs. The sequences of 115 PyV LTAs were retrieved and scanned for the presence of putative monopartite or bipartite cNLSs. (A) The percentage of LTAs from PyVs classified in the indicated genus bearing a cNLS (yellow) or not bearing a cNLS (purple) is indicated. (B) The percentage of PyV LTAs bearing a cNLS downstream of the LXCXE motif (yellow) or in a different position (purple) is shown. (C) The percentage of PyV LTAs bearing a monopartite (yellow) or bipartite (purple) cNLS downstream of the LXCXE motif is indicated. (D) Sequences were phylogenetically grouped by ClustalW and MEGA X. The evolutionary history was inferred using the Neighbor‐Joining method. The optimal tree with the sum of branch length = 24.63317861 is shown. The percentage of replicate trees in which the associated taxa clustered together in the bootstrap test (500 replicates) are shown next to the branches. The evolutionary distances were computed using the Poisson correction method and are in units of the number of aa substitutions per site. Each PyV LTA is indicated as a circle, colored according to the viral genus. Filled circles indicate the presence of a cNLS downstream of the LXCXE motif, similar to SV40 LTA cNLS (cNLS between LXCXE motif and OBD), whereas empty circles indicate a cNLS elsewhere (no cNLS between LXCXE motif and OBD); an asterisk indicates a bipartite cNLS (bipartite cNLS between LXCXE motif and OBD); a small red circle indicates that no cNLS has been identified (no cNLS). cNLS, classical nuclear localization signal; LTA, large tumor antigen.

Our findings that cNLSs of HPyVs are extremely heterogenous in terms of composition and activity prompted us to extend such analysis to all known PyVs. We therefore bioinformatically analyzed the sequences of LTAs from all known PyVs by clustalW, to generate phylogenetic trees, and cNLS mapper, to identify putative cNLSs. Since software detection of cNLSs can be inaccurate for the detection of weak cNLSs (such as in the case of HPyV7) or bipartite cNLSs (such as in the case of KIPyV, STLPyV, WUPyV, MCPyV, and MWPyV), all sequences were also visually inspected for clusters of basic aas. Only 6 LTAs out of 115 (5%) do not contain a putative cNLS (Figure 9A, Table S8), highlighting the importance of the IMPα/β pathway for LTA nuclear import. Intriguingly, most LTAs without a putative cNLS belong to the *Gammapolyomavirus* genus, which comprises viruses infecting birds (Figure 9D). In all cases, cNLSs were preferentially located between the LXCXE motif and OBD, similar to that observed for SV40 LTA and the HPyV LTAs studied here, the only exceptions being represented by *Gammapolyomaviruses*, infecting birds, and unclassified PyVs, infecting fishes, which do not possess putative cNLSs in such positions (Figure 9B, D). Surprisingly, our analysis revealed that 45% of cNLSs located between the LXCXE motif and OBD are potentially bipartite (Figure 9C). Such cNLS configuration was particularly enriched in alpha (53% of cases) and delta (>60% of cases) PyVs. Bipartite cNLSs are present in clusters of phylogenetically distinct viruses, thus suggesting they have been generated by multiple independent events during evolution of PyVs, possibly reflecting a process of virus‐host adaptation (Figure 9D).

## 3 DISCUSSION

Here we have comprehensively analyzed the nuclear transport process of the LTAs from all known HPyVs. Despite the 127‐P**KKKRK**V‐132 sequence from SV40 LTA being the first NLS identified (Kalderon, Richardson, et al., 1984; Kalderon, Roberts, et al., 1984), very little is known regarding the nuclear import of other family members. Our comprehensive functional, biochemical, and structural analyses revealed several important features, with novel implications for the biology and evolution of both PyVs and IMPα/β‐dependent NLSs, paving the way for understanding how different types of cNLSs might have evolved.

### 3.1 cNLSs are essential for IMPα/β‐mediated nuclear import of HPyV LTAs

Similar to what has previously been demonstrated for SV40 (Conti et al., 1998), LTAs from all HPyVs contain a functional cNLS located between the LXCXE motif and the OBD (Figure 2) which is recognized by IMPα (Figures 4 and 5), implying that HPyV LTAs are translocated into the nucleus by the IMPα/β pathway. Indeed, we demonstrate here for the first time that nuclear import of full‐length LTAs from MCPyV, KIPyV, STLPyV, MWPyV, and HPyV7 is abrogated by the well‐known IMPα/β competitive inhibitor Bimax2 (Figure 3), which is capable of sequestering IMPα by simultaneously binding its minor and major NLS binding sites in the absence of IMPβ (Kosugi et al., 2008). Furthermore, substitution of key basic residues in full‐length LTAs from MCPyV, KIPyV, STLPyV, MWPyV, and HPyV7 completely abrogated nuclear import, proving that the NLSs identified here are the major determinants of nuclear import (Figures 7 and 8).

### 3.2 Conservation of cNLSs among all known PyV LTAs

95% of LTAs from all known PyV species contain a putative cNLS (Figure 9A), which in more than 80% of cases is located immediately downstream of the LXCXE motif, in a similar position to the SV40 LTA cNLS (Figure 2A,B, Figure 9B). This implies that most PyVs rely on IMPα/β to ensure proper translocation of their LTAs to the nucleus. This has potential implications for antiviral therapy, since the FDA approved antiparasitic drug Ivermectin, a potent inhibitor of the IMPα/β pathway, has been shown to inhibit BKPyV replication in cell culture (Bennett et al., 2015). However, only 55% of LTAs from *Gammapolyomaviruses* possess a putative cNLS (Figure 9A), which are located in different positions within the viral protein (Figure 9B). This is surprising as birds possess functional IMPα orthologues, although with distinct properties (Pumroy et al., 2015), and is likely the consequence of ancient divergence between mammals and birds (Hedges et al., 1996).

### 3.3 Structural and functional heterogenicity among PyV LTA cNLS activity

Intriguingly, our bioinformatics analysis revealed that 45% of PyV LTAs with a cNLS located immediately downstream of the LXCXE motif possess a putative bipartite cNLS (Figure 9C). Indeed, the most striking finding of our study is represented by the high cNLS structural and functional heterogenicity observed in LTAs from the 14 HPyVs characterized here. Six possess a single, functional monopartite cNLS (BKPyV, JCPyV, HPyV6, HPyV7, HPyV9, and TSPyV LTAs). Two possess a bipartite cNLS (STLPyV and KIPyV LTAs). The others (MWPyV, WUPyV, MCPyV, and possibly HPyV12 and LIPyV LTAs) present more than one closely located cNLS (Figure 2B, Figure S1). These can function independently as a monopartite cNLS (Figure 2D,E), but preferentially work as a bipartite cNLS, with the upstream and downstream stretches of basic aas binding to the IMPα minor and major binding sites, respectively (Figure 4), in a similar manner to that reported for the nucleoplasmin bipartite cNLS (Conti & Kuriyan, 2000). Most monopartite cNLSs are fully functional, perfectly fit to the consensus (K‐K/R‐X‐K/R, Figure 1, Figure S1), and can be expected to bind to the IMPα major binding site in an extended conformation, similar to JCPyV LTA (Figure 4), SV40 LTA, and several other monopartite cNLSs (Alvisi et al., 2023; Conti et al., 1998; Smith et al., 2018). However, HPyV7 LTA contains an extremely weak cNLS (143‐PP**K**Q**KK**PN‐152), which does not fit the cNLS consensus but is still capable of partially relocating GFP to the nucleus (Figure 2Dl,E) and weakly binding IMPα3ΔIBB in vitro (Figure S5B). Attempts to solve the crystal structure of the IMPα2ΔIBB:HPyV7 LTA cNLS complex proved unsuccessful, likely due to inefficient binding.

### 3.4 Binding properties of HPyV LTA cNLSs with IMPα isoforms

Overall, cNLS activity is highly variable, with bipartite cNLSs (Fn/c between 6.3 and 32.2) being more active in terms of nuclear transport when fused to GFP compared to monopartite cNLSs (Fn/c between 1.6 and 10.9). The functional variability in the nuclear targeting activity of the HPyV LTA cNLSs described here is likely the consequence of differences in IMPα binding properties, as observed in EMSA and FP experiments (Figure 5, Figure S4), consistent with the notion that nuclear transport efficiency depends on IMPα:cNLS binding affinity (Hodel et al., 2006; Smith et al., 2018). HPyV LTA bipartite cNLSs bound IMP αΔIBB with higher affinity compared to monopartite cNLSs (Table S6), as was expected from what has previously been shown in the literature (Hoad et al., 2023; Hodel et al., 2001). Spacing between the basic aa stretches, known as the linker region, is also variable, ranging from 11 aas for MWPyV LTA to 32 aas for HPyV12 LTA (Figure 4A). Although we did not test the functionality of the HPyV12 LTA bipartite NLS, our structural analysis of STLPyV, KIPyV, WUPyV, and MCPyV LTA cNLSs clearly shows that different linker lengths (range 11–21 aas) are allowed in bipartite cNLSs (Figure 4B). This is consistent with recent studies demonstrating that porcine adeno‐associated virus capsid protein possesses a bipartite NLS with a 26 aa linker (Hoad et al., 2023), and that distantly located NLSs in SOX2 make a contiguous interface with IMPα3 (Jagga et al., 2021). Furthermore, all tested HPyV LTA cNLSs exhibited IMPα isoform specificity, with those from WUPyV and JCPyV binding preferentially to IMPα1ΔIBB, and that from KIPyV binding preferentially to IMPα7ΔIBB. Such specificity has already been demonstrated for several other cellular and viral proteins (Dohner et al., 2018; Jagga et al., 2021; Ninpan et al., 2016; Pumroy et al., 2015), and since IMPα isoforms are differently expressed in human tissues, this has potential implications for our understanding of HPyVs tropism (Lanford & Butel, 1980b, 1984). Featuring a weak cNLS, HPyV7 LTA poorly accumulated in the cell nucleus (Figure 3A,B), and selectively bound to IMPα3ΔIBB (Figure S5B). Importantly, the efficiency of nuclear transport appeared to be directly linked to protein expression levels, with lower expression levels associated with higher nuclear accumulation (data not shown). Taken together, such data suggest that IMPα3 levels may be limiting for HPyV7 LTA nuclear import.

### 3.5 A hybrid NLS in MCPyV LTA

**FIGURE 10:**
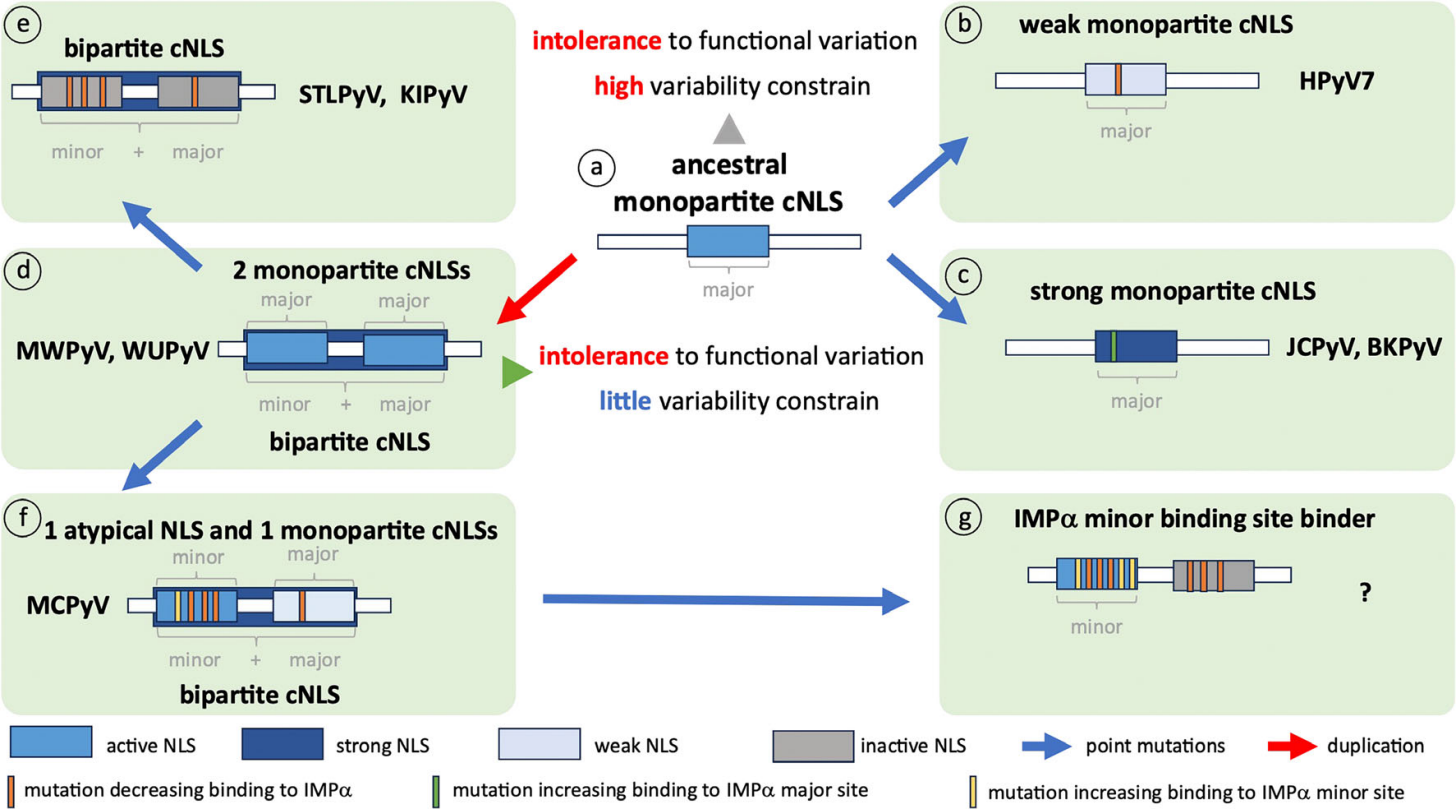
A model for evolution of different classes of cNLSs from an ancestral monopartite cNLS in PyV LTAs. An ancestral monopartite cNLS needs to functionally interact with the IMPα major binding site to mediate LTA nuclear import and viral replication, and is therefore subjected to (gray arrowhead) intolerance to functional variation and high variability constrain (a). Point mutations (blue arrows) can introduce aa substitutions within the cNLS sequence that can either decrease (orange vertical bars) the interaction with IMPα at the major site, resulting in a weaker cNLS such as those described here for HPyV7 LTA (b), or increase (green vertical bars) the interaction with IMPα at the major site, resulting in a stronger cNLS such as that described here for JCPyV LTA, or described elsewhere for BKPyV LTA, which is identical to SV40 LTA (c). Duplication of the monopartite sequence during viral genome replication (red arrow) would generate two monopartite cNLSs, able to either individually interact with the IMPα major binding site, or simultaneously bind to the IMPα minor and major binding sites, as described here for MWPyV and WUPyV LTAs (d). In such a scenario (green arrowhead), the sequence would still be subjected to intolerance to functional variation, but the aa variability constrain would be relaxed, allowing the sequences to accumulate mutations. This could cause aa substitutions impairing the ability of the two cNLSs to interact individually with the IMPα major site (orange vertical bars). However, this would still allow simultaneous binding to the IMPα minor and major binding sites, resulting in the selection of bipartite cNLSs, such as those described here for STLPyV and KIPyV LTAs (e). Alternatively, substitutions in the upstream cNLS could optimize interaction with the IMPα minor binding site (yellow vertical bars), while substitutions in the downstream cNLS could impair autonomous binding at the IMPα major site (orange vertical bars), resulting in a hybrid NLS such as that described here for MCPyV LTA, which could function as a bipartite cNLS, but, in the case of further deletion of the downstream cNLS, could function as a minor site‐specific atypical cNLS (f). Further mutations in the upstream cNLS, optimizing binding at the minor site (yellow vertical bars), and in the downstream cNLS, preventing binding at the major site (orange bars), would then result in selection of an atypical cNLS, exclusively binding at the IMPα minor site (g). cNLS, classical nuclear localization signal; LTA, large tumor antigen.

MCPyV LTA is unique in that it contains two cNLSs (Figure 2) which preferentially work as a bipartite cNLS (Figure 8), with the upstream NLSm binding the minor and the downstream NLSct binding the major IMPα binding sites, respectively (Figure 4Bf). However, in the absence of NLSct, NLSm is still functional (Figure 2) and can directly bind the IMPα minor site with micromolar affinity (Figure 8H, Figure S6C). Such a finding is of extraordinary importance for both HPyV pathophysiology and cell biology. It suggests that such hybrid cNLSs offer maximum flexibility for nuclear import of MCPyV LTA. Interestingly, the MCPyV genome is integrated in host cell chromosomes in more than 80% of cases of MCC, a rare and very aggressive form of skin cancer (Becker et al., 2017). Integration of the viral genome usually occurs upstream of the coding sequence for the LTA helicase domain coding sequence, resulting in a defective viral genome, unable to replicate but still able to sustain cellular proliferation by binding to the Rb protein (Houben et al., 2010). In a considerable number of such clones, integration occurs between the NLSm and NLSct coding sequences, generating a truncated LTA fragment whereby NLSct (and thus the bipartite NLS) is destroyed, but NLSm is preserved (Ortiz et al., 2021). Therefore, while full‐length MCPyV LTA can be imported to the nucleus in high levels and via multiple IMPα isoforms using a bipartite cNLS, truncated LTA fragments can still interact with the IMPα minor binding site through NLSm (Figure 8H, Figure S6) and be actively transported into the nucleus (Figure 8E–G). In addition, this represents the first evidence of how minor site cNLSs could have evolved from duplication of monopartite cNLSs and by subsequently acquiring mutations that confer specificity for the IMPα minor site (Figure 10).

### 3.6 A tentative model for evolution of the three types of cNLSs

One of the most striking findings of our study is that several PyVs evolved bipartite cNLSs in their LTA coding sequences independently (Figure 9D). It is tempting to speculate that this is the result of several independent duplication events of an original monopartite cNLS during the evolution of PyVs. Indeed, not only the basic residues but also the T residues which are the target of cdc2 phosphorylation are duplicated (Figure S1). In this scenario, the different cNLSs characterized here would have originated by mutation and duplication of a major site‐interacting ancestral monopartite cNLS (Figure 10A). Given the importance of LTA nuclear targeting for HPyV replication, such sequences would have been subjected to intolerance to functional variation (i.e., IMPα binding must be preserved) and high sequence variability constrain (Figure 10, *gray arrowhead*). Indeed, gross mutations of the cNLS aa sequence strongly impairing the IMPα:cNLS interaction would not be compatible with viral replication (Lanford & Butel, 1984). However, such ancestral cNLSs would still be able to tolerate subtle aa changes (Figure 10, *blue arrows*), modulating the NLS:IMPα interaction affinity. Such mutations may therefore cause either a decrease in NLS:IMPα interaction affinity (Figure 10B, *orange vertical bars*), resulting in a weak monopartite cNLS such as that identified for HPyV7 LTA, or an increase in NLS:IMPα interaction affinity (Figure 10C, *green vertical bars*), resulting in a stronger monopartite cNLS such as those present in JCPyV and BKPyV LTAs. Conversely, a duplication event involving an ancestral monopartite cNLS (Figure 10, *red arrow*) would have generated two closely located cNLSs, capable of binding IMPα either autonomously at the major binding site, or as a bipartite cNLS at the major and minor binding sites simultaneously (Figure 10D). Such a scenario is exemplified by MWPyV and WUPyV LTAs, whose two cNLSs are still functional independent of each other, but together act as a bipartite cNLS. Such sequences would be subjected to much lower variability constrain compared to an individual monopartite cNLS, despite the constant intolerance to functional variation (Figure 10, *green arrowhead*). Indeed, both the upstream and downstream cNLSs could tolerate mutations impairing their ability to bind IMPα autonomously (Figure 10E, *gray orange bars*), preserving their ability to simultaneously bind to the minor and major binding sites as a bona fide bipartite cNLS. This scenario is exemplified by STLPyV and KIPyV LTA bipartite cNLSs, where the upstream cNLS has lost most key features of a monopartite cNLS (Figure 10E). In some cases, the upstream cNLS might acquire mutations which strengthen the interaction at the minor binding site (Figure 10F, *yellow vertical bars*) but weaken the interaction at the major binding site (Figure 10F, *orange vertical bars*). This can result in a hybrid NLS, which can function as a bipartite cNLS whereby the upstream and downstream cNLSs bind simultaneously to the minor and major IMPα binding sites, while the upstream cNLS can functionally interact with the IMPα minor site in the absence of the downstream cNLS, as seen in the case of MCPyV LTA (Figure 10F). The sequence could further accumulate mutations in the downstream cNLS, impairing its ability to interact with the IMPα major site, even in the presence of the upstream cNLS. This in turn could further accumulate mutations, strengthening its ability to independently interact with the IMPα minor binding site. Such a process, which did not apparently occur in any HPyV LTAs, could therefore lead to the evolution of minor site‐specific NLSs (Figure 10G). Nonetheless, considering the limited knowledge about PyV evolution and the fact that it likely includes multiple species jumps, lineage duplications, and recombination events (Torres, 2020), only additional data and fine molecular clock analyses will be able to confirm such hypotheses, resolving the time‐scale and the order of the events proposed in Figure 10.

### 3.7 Conclusions

In summary, we have provided the first structural and functional comparative analysis of cNLSs across a viral family, and identified important differences across all HPyV LTAs, with implications for our understanding of viral tropism determinants and the development of antiviral approaches. Since the different HPyV LTA NLSs can interact specifically with either IMPα major, minor, or both binding sites, typical of monopartite, atypical, and bipartite cNLSs, respectively, our study could provide a starting point for furthering our understanding of IMPα‐binding NLS evolution.

## 4 MATERIALS AND METHODS

### 4.1 Bioinformatics

The sequences for all HPyVs LTAs were retrieved from UniProtKB, using the following UniProt codes: BKPyV, P14999; JCPyV, P03072; KIPyV, P0DOI6; WUPyV, A5HBG1; MCPyV, A0A173M1N7; HPyV6, D6QWG6; HPyV7, D6QWI6; TSPyV, E2ESL8; HPyV9, E9NQ91; MWPyV, A0A159B681; STLPyV, L7RFY1; HPyV12, M4STH5; NJPyV, A0A024B6C0; LIPyV, A0A4V1I211. The coding sequences of the two LTAs used as positive controls were similarly retrieved: MPyV with UniProt code P03073, and SV40 with UniProt code P03070. Sequences were aligned with Clustal W (Thompson et al., 1994 **)**. cNLS sequences were identified by cNLS mapper (Kosugi, Hasebe, Tomita, & Yanagawa, 2009), and by visual inspection to identify sequences potentially matching the cNLS consensus (Smith et al., 2018). The sequences of HPyV7 clinical isolates containing full‐length LTAs were retrieved from GeneBank and aligned with Clustal W (Thompson et al., 1994). For phylogenic analysis of all PyV LTAs, the sequences of LTAs from 115 PyVs were retrieved from UniProt (Table S8). The evolutionary history was inferred using the Neighbor‐Joining method (Saitou & Nei, 1987). The percentage of replicate trees in which the associated taxa clustered together in the bootstrap test (500 replicates) were shown next to the branches (Felsenstein, 1985). The evolutionary distances were computed using the Poisson correction method and expressed in units of the number of aa substitutions per site. This analysis involved 115 aa sequences. All ambiguous positions were removed for each sequence pair (pairwise deletion option). There were a total of 1961 positions in the final dataset. Evolutionary analyses were conducted in MEGA X (Kumar et al., 2018).

### 4.2 Plasmids

Plasmids pEGFP‐N1‐H1E, pGFP‐UL44, and pGFP‐UL44‐C2N(405–433), encoding empty vectors or control GFP fusion proteins localizing to the nucleus via different pathways, were described previously (Alvisi et al., 2005, 2006, 2008). Mammalian expression construct pcLT206‐eGFP (Liu et al., 2011), mediating expression of full‐length LTA from MCPyV, was kindly provided by Patrick Moore (Pittsburgh, USA). Plasmid mcherry‐Bimax2, encoding for a competitive inhibitor of the IMPα/β nuclear import pathway (Tsujii et al., 2015), was a generous gift from Yoshihiro Yoneda and Masahiro Oka (Osaka, Japan). Mammalian expression plasmids encoding for cNLS fused to the C‐terminus of cycle 3 GFP were generated by annealing appropriate oligonucleotide pairs in vector pcDNA3.1/NT‐GFP‐TOPO® (Thermofisher Scientific, Monza, Italy). Mammalian expression plasmids encoding for YFP fusion proteins were generated using Gateway™ technology by cloning appropriate cDNAs into pDNR207 by PCR via BP reactions and subsequently transferring them to pDESTntYFP via LR reactions, as previously described (Sinigalia et al., 2008). Plasmids encoding HPyV7, STLPyV, KIPyV, and MWPyV LTAs fused to the N‐terminus of GFP were synthesized (Vector Builder, Neu‐Isenburg, Germany and BioFab Research, Rome, Italy), and substitution derivatives thereof were generated using the Quikchange mutagenesis kit (Agilent Technologies, Cernusco sul Naviglio (MI), Italy) according to the manufacturer's recommendation and using appropriate oligonucleotide pairs. All plasmids were verified by Sanger sequencing (BMR Genomics, Padova, Italy). Lists of all oligonucleotides and plasmids used in this study are available in Tables S9 and S10, respectively.

### 4.3 Cell culture

HEK293A cells were maintained in Dulbecco's modified Eagle's medium (DMEM) supplemented with 10% (v/v) fetal bovine serum (FBS), 50 U/mL penicillin, 50 U/mL streptomycin, and 2 mM L‐glutamine (all from Thermofisher Scientific, Monza, Italy), and passaged when confluent.

### 4.4 Confocal laser scanning microscopy/image analysis

HEK293A cells were seeded onto glass coverslips in a 24‐well plate (4 × 10^4^ cells/well) and the next day transfected with appropriate amounts of expression constructs (range 5–250 ng) using Lipofectamine 2000 (Thermofisher Scientific, Monza, Italy) as previously described (Trevisan et al., 2018). At 48 h post‐transfection (p.t.), cells were incubated for 30 min with DRAQ5 (1:5000 in DMEM, no phenol red), washed with PHEM 1x (60 mM PIPES, 25 mM HEPES, 10 mM EGTA, and 4 mM MgSO_4_), and fixed with paraformaldehyde/PHEM 3% (v/v) for 10 min. Following three washes with PHEM 1x and one wash with milliQ water, coverslips were mounted on glass slides with Fluoromount G (Southern Biotech, Birmingham, AL, USA). Subcellular localization of fusion proteins was analyzed using a Leica Nikon A1 confocal laser scanning microscope (Nikon, Tokio, Japan) equipped with a 60x oil immersion objective, as described previously (Smith et al., 2018). Levels of nuclear accumulation of proteins of interest were determined using the Fiji public domain software (https://doi.org/10.1038/nmeth.2019) from single cell measurements of both the nuclear (Fn) and cytoplasmic (Fc) fluorescence, subsequent to the subtraction of fluorescence due to autofluorescence/background fluorescence, as described previously (Alvisi et al., 2018). Data were statistically analyzed by performing either student's t test or one‐way ANOVA using Prism 9 (GraphPad) software.

### 4.5 Protein purification

Recombinant IMPαΔIBB proteins were produced for structural analysis and assays to elucidate the binding profiles with PyV LTAs. For this, plasmid constructs encoding IMPα isoforms with an N‐terminal truncation removing the autoinhibitory IBB domain (ΔIBB) were synthesized (GenScript Biotech, Singapore). Codon optimized genes were designed with an N‐terminal 6xHis tag and TEV protease cleavage site and were cloned into the BamHI site of pET30a (GenScript Biotech, Singapore). Accession numbers include IMPα1 (UniProtKB: P52292, aa 70–529), IMPα3 (UniProtKB: O00629, aa 64–521), IMPα5 (UniProtKB: P52294, aa 73–538), and IMPα7 (UniProtKB: O60684, aa 73–536) for the human IMPα proteins, as well as IMPα2 (UniProtKB: P52293, aa 70–529) for the mouse homolog of IMPα1. For protein expression, the plasmids were transformed via heat shock into BL21(DE3)pLysS *E. coli* cells (Thermofisher Scientific, Monza, Italy) and grown in 1 L baffled flasks at room temperature using auto‐induction methods for 24–48 h (Studier, 2005). Following two cell pellet freeze–thaw cycles and treatment with lysozyme and DNAse, the clarified cell lysate was purified using a 5 mL nickel affinity HisTrap HP column (Cytiva, Marlborough, USA) with wash buffer (50 mM phosphate buffer, 300 mM NaCl, 20 mM imidazole, pH 8.0) and a 10‐column volume gradient of elution buffer (50 mM phosphate buffer, 300 mM NaCl, 500 mM imidazole, pH 8.0). Following this, the 6xHis tag was cleaved using TEV protease incubated overnight at 4°C, and then further purified via gel filtration using a Superdex 200 pg 26/600 column (Cytiva, Marlborough, USA) and tris‐buffered saline (50 mM tris, 125 mM sodium chloride, pH 8.0). The IMPα2ΔIBB protein did not contain a TEV cleavage site and was purified by gel filtration with its 6xHis tag. Peak fractions were assessed for purity using SDS‐PAGE, then pooled and concentrated using 10 kDa cutoff centrifugal filters (Merck Millipore, Milan, Italy). All samples were aliquoted and stored at −80°C until further use.

### 4.6 Peptide synthesis

Peptides corresponding to the cNLS regions of each PyV were synthesized for use in crystallography and binding assays. An automated CEM Liberty Blue solid phase peptide synthesizer generated each peptide with an added N‐terminal fluorescein isothiocyanate (FITC) tag. The reaction was performed using DIC and Oxyma coupling reagent and Fmoc deprotection with 20% (v/v) piperidine/DMF solution, all at 90°C. Following the final coupling, peptides were cleaved from the resin using a solution of TFA/TIPS/H_2_O (95/2.5/2.5) for 3 h. Purification was performed with HPLC and the peptides lyophilized to powder. Prior to use, peptide stock solutions were resolubilized to 10 mM concentration, aliquoted, and stored at −20°C until use. All peptide sequences are listed in Table S11.

### 4.7 Electrophoresis mobility shift assays

For analysis of binding in a native gel, EMSAs were performed using IMPαΔIBB proteins ± FITC‐cNLS peptides, as described previously (Wagstaff et al., 2005). Briefly, a 1.5% agarose gel in TB buffer (0.45 mM tris, 0.45 mM boric acid, pH 8.5) was loaded with recombinant IMPαΔIBBs ± FITC‐cNLS peptides and run at 70 V for 2 h. Two images were acquired for analysis, one using a UV filter to detect fluorescent peptides, and another using visible light to observe Coomassie‐stained protein bands.

### 4.8 Fluorescence polarization (FP) assays

FP was used to quantify binding affinities of the HPyV LTA cNLSs for IMPαΔIBB proteins. Two‐fold dilutions of recombinant IMPαΔIBB proteins were titrated into black Fluotrac microplates (Greiner Bio‐One, Kremsmünster, Austria) and FITC‐cNLS peptides were added to each well before making up to a total volume of 200 μL with tris‐buffered saline. The gain adjustment was made using a well with no FITC‐NLS peptide. The CLARIOstar Plus (BMG Labtech, Mornington, Australia) plate reader measured FP values, with each assay repeated in triplicate. Binding curves to calculate *K*_*d*_ and *B*_max_ values were generated with the one site specific binding least square fit function of Prism 9 (GraphPad) software.

### 4.9 Crystallization of cNLS peptides complexed with IMPα2ΔIBB

X‐ray crystallography was employed to characterize HPyV LTA cNLSs bound to IMPα2ΔIBB. Crystal screening was performed with hanging drop vapor diffusion and a range of known precipitant conditions (0.50–0.85 M sodium citrate, 0.10 M HEPES pH 6.5/7.0/7.5, 0.01 M DTT). Each well contained 300 μL of precipitant and each hanging drop comprised 3 μL total volume. The hanging drops either contained a 3:1 molar ratio of IMPα2ΔIBB:FITC‐cNLS peptide or IMPα2ΔIBB only. For the latter, pre‐grown apo IMPα2ΔIBB crystals were soaked with FITC‐cNLS peptide just prior to flash freezing in liquid nitrogen. Following cryo‐protection in precipitant condition plus 20% glycerol, harvesting, and flash freezing, the crystals were diffracted at the Australian Synchrotron on the MX1 (Cowieson et al., 2015) or MX2 (Aragao et al., 2018) beamlines. Diffraction data were processed using iMosflm (Battye et al., 2011) or XDS auto‐processing (Kabsch, 2010), merged, scaled, and *R*_free_ selected using Aimless (Evans & Murshudov, 2013), phased by molecular replacement (McCoy et al., 2007), modeled in Coot (Emsley & Cowtan, 2004), then refined using Phenix (Adams et al., 2010). All models underwent rounds of iterative model building and refinement until final structures were validated and deposited in the Protein Data Bank (PDB). All crystallization conditions and collection and refinement statistics are listed in Tables S4 and S5. Protein interface interactions used in the structure figures were calculated using PDBePISA (Krissinel & Henrick, 2007), with hydrogens removed in the PDB input models.

## AUTHOR CONTRIBUTIONS

**Gualtiero Alvisi:** Conceptualization; data curation; formal analysis; visualization; writing – original draft; writing – review and editing; project administration; supervision; investigation; methodology; validation; funding acquisition. **Emily M. Cross:** Data curation; formal analysis; methodology; writing – original draft; visualization; investigation. **Nasim Akbari:** Data curation; methodology; investigation; formal analysis. **Hanieh Ghassabian:** Data curation; formal analysis; methodology; investigation. **Mikayla Hoad:** Data curation; formal analysis; methodology; investigation. **Silvia Pavan:** Methodology. **Daryl Ariawan:** Methodology. **Camilla M. Donnelly:** Data curation; methodology; formal analysis. **Enrico Lavezzo:** Formal analysis; software. **Gayle F. Petersen:** Data curation; methodology; formal analysis; writing – review and editing. **Jade Forwood:** Supervision; funding acquisition; writing – review and editing.

## Supporting information


**FIGURE S1:** Sequence alignments of HPyV LTA NLS region.
**FIGURE S2:** Correlation between cNLS mapper score and cNLS activity.
**FIGURE S3:** Nuclear accumulation of HPyV LTAs is dependent on the IMPα/β heterodimer.
**FIGURE S4:** EMSAs between LTA cNLS peptides and IMPαΔIBB isoforms.
**FIGURE S5:** HPyV7 LTA cNLS weakly binds IMPα3ΔIBB.
**FIGURE S6:** Binding assays for MCPyV cNLS regions and mutants.
**FIGURE S7:** Superposition of MCPyV LTA NLSm with minor site‐specific binder RNA helicase II/Gu⍺ NLS.

**TABLE S1:** Summary of cNLSs identified on HPyVs LTAs.
**TABLE S2:** Identification and functional validation of putative cNLS in LTAs from HPyVs.
**TABLE S3:** Nuclear localization of LTAs from several HPyVs is inhibited by Bimax2.
**TABLE S4:** Crystallization information and data processing statistics for IMP⍺2ΔIBB:cNLS structures.
**TABLE S5:** Crystallization information and data processing statistics for MCPyV LTA cNLS:IMP⍺2ΔIBB mutant structures.
**TABLE S6:** LTA cNLSs bind with different affinity to IMPα isoforms.
**TABLE S7:** Effect of specific amino acid substitutions on nuclear accumulation of LTAs from the indicated HPyVs.
**TABLE S8:** List of main features of all known LTAs and summary NLS statistics.
**TABLE S9:** Oligonucleotides used in this study.
**TABLE S10:** Plasmids used in this study.
**TABLE S11:** Summary of peptides synthesized for binding assays and co‐crystallization with IMP⍺2ΔIBB.

## Data Availability

The data underlying this article, as well as all plasmid expression construct generated in this study will be shared upon reasonable request to the corresponding author.
