# Supplementary material for: A functional and structural comparative analysis of large tumor antigens reveals evolution of different importin α‐dependent nuclear localization signals
Source: Protein Sci. 2024 Feb 1;33(2):e4876. doi: 10.1002/pro.4876 (PMC10807245; doi:10.1002/pro.4876)
Supplement: Supplementary file 1 — FIGURE S1: Sequence alignments of HPyV LTA NLS region. FIGURE S2: Correlation between cNLS mapper score and cNLS activity. FIGURE S3: Nuclear accumulation of HPyV LTAs is dependent on the IMPα/β heterodimer. FIGURE S4: EMSAs between LTA cNLS peptides and IMPαΔIBB isoforms. FIGURE S5: HPyV7 LTA cNLS weakly binds IMPα3ΔIBB. FIGURE S6: Binding assays for MCPyV cNLS regions and mutants. FIGURE S7: Superposition of MCPyV LTA NLSm with minor site‐specific binder RNA helicase II/Gu⍺ NLS. [file PRO-33-e4876-s002.pptx]

## Slide 1
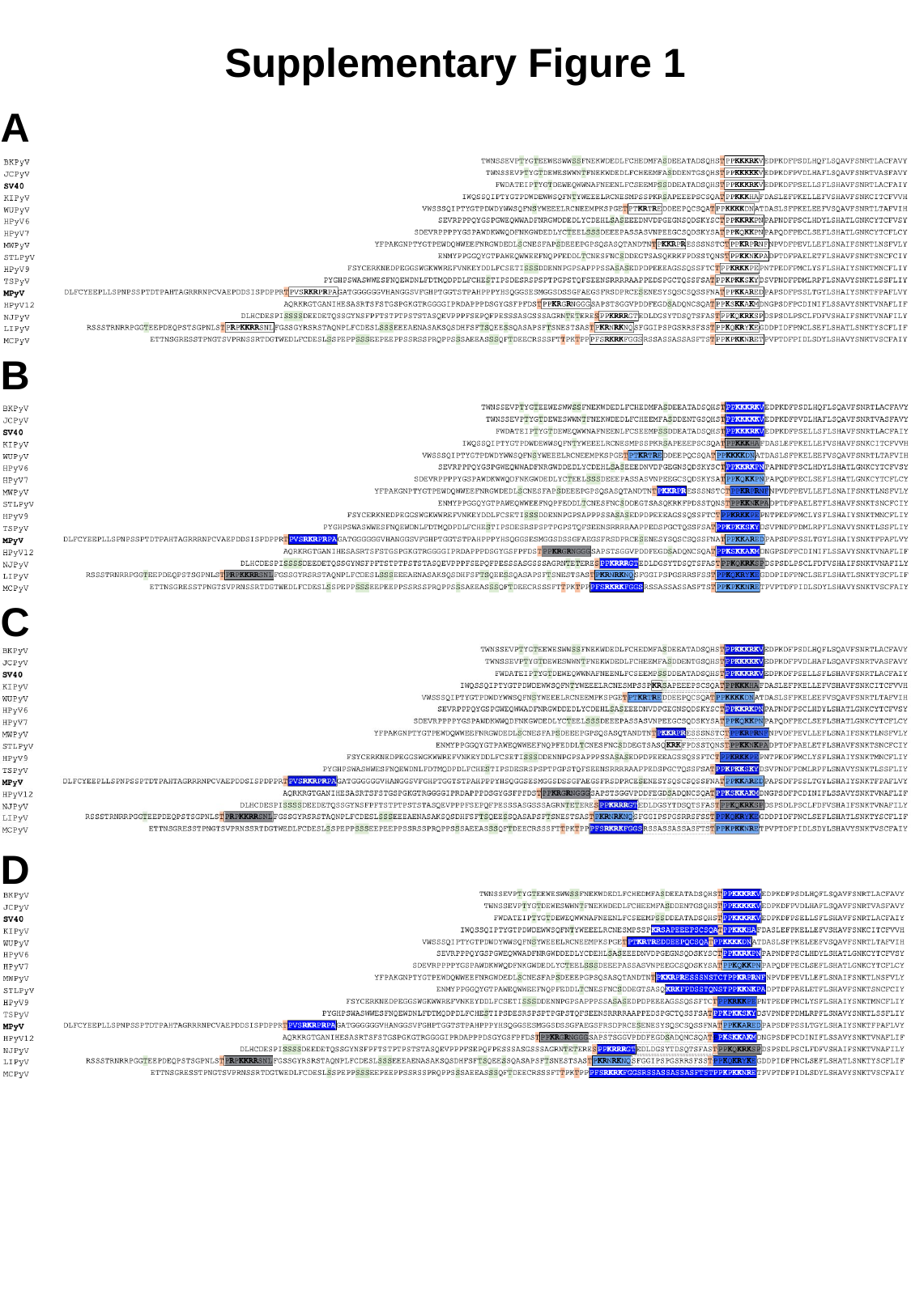

Supplementary Figure 1
A
B
C
D

## Slide 2
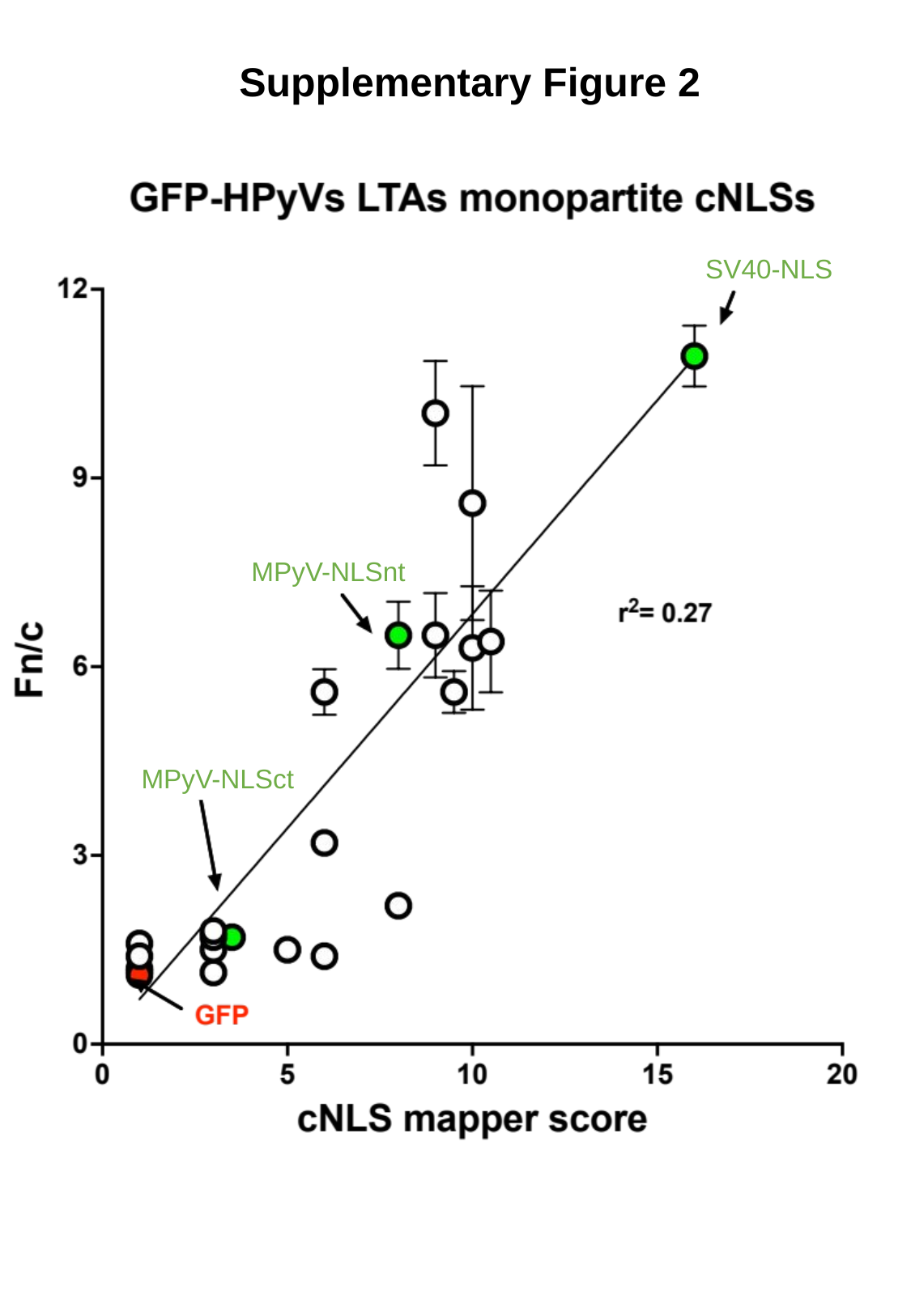

Supplementary Figure 2
SV40-NLS
MPyV-NLSnt
MPyV-NLSct

## Slide 3
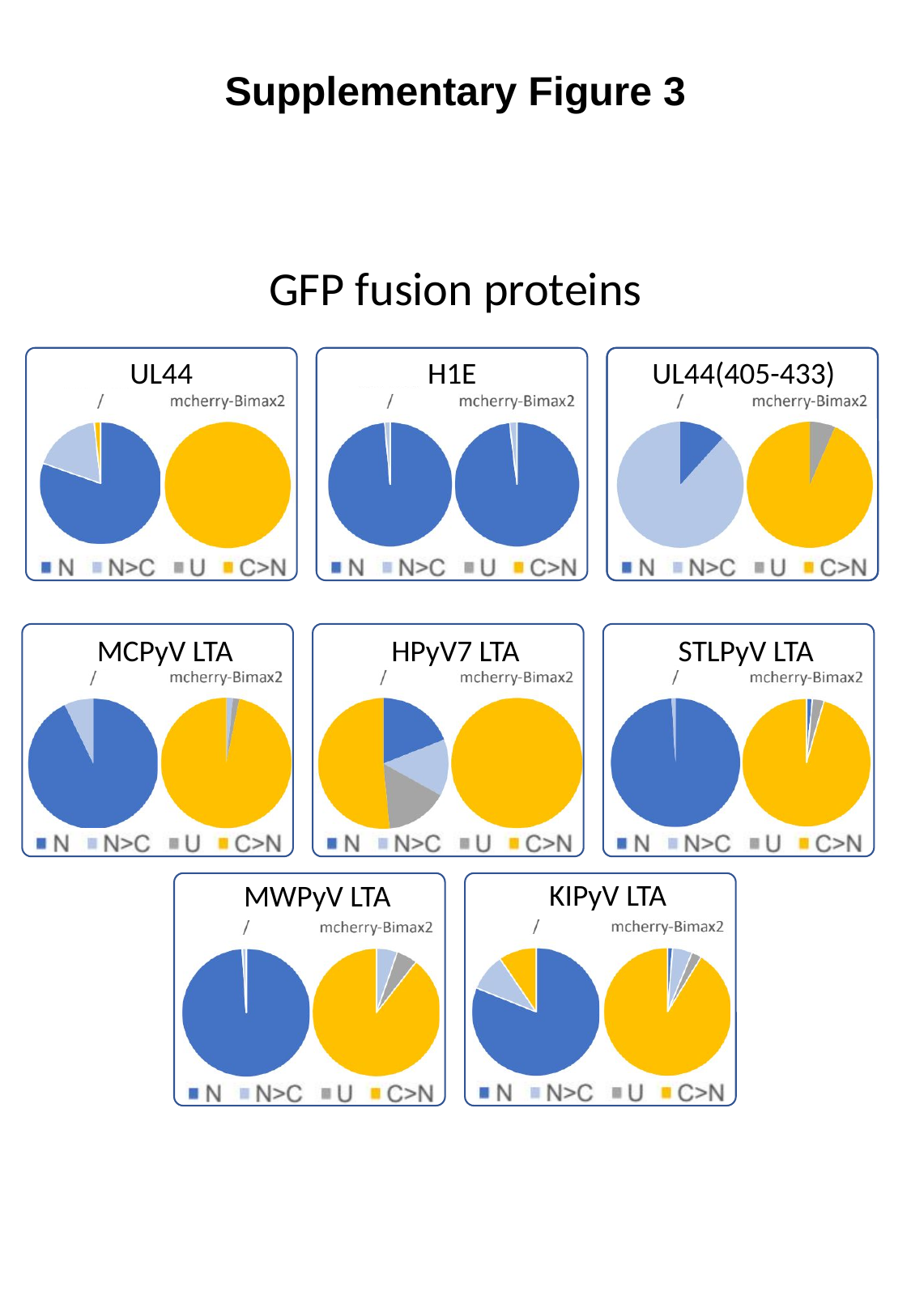

Supplementary Figure 3
GFP fusion proteins
UL44
H1E
UL44(405-433)
MCPyV LTA
HPyV7 LTA
STLPyV LTA
KIPyV LTA
MWPyV LTA

## Slide 4
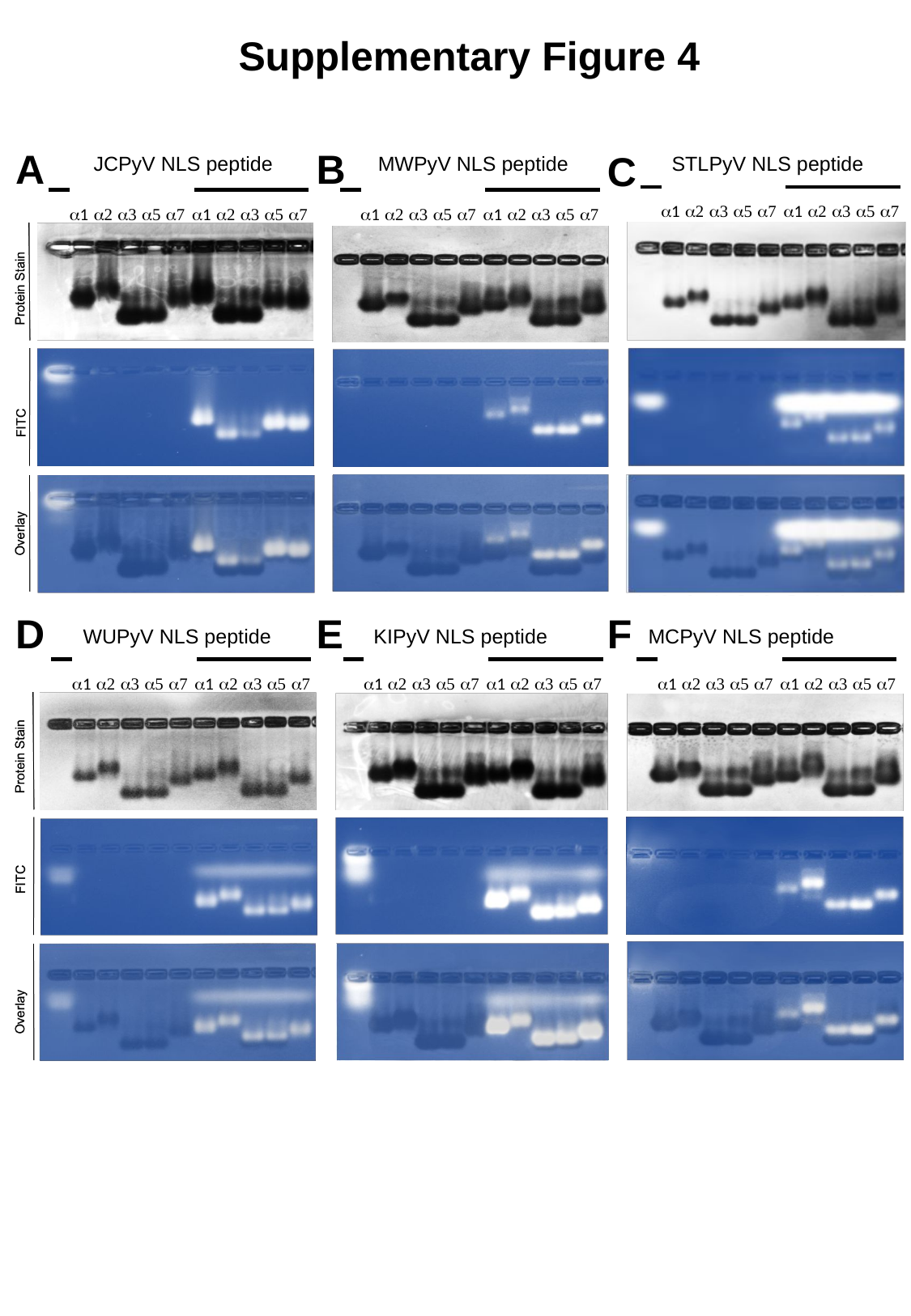

Supplementary Figure 4
A
B
C
JCPyV NLS peptide
MWPyV NLS peptide
STLPyV NLS peptide
a1
a2
a3
a5
a7
a1
a2
a3
a5
a7
a1
a2
a3
a5
a7
a1
a2
a3
a5
a7
a1
a2
a3
a5
a7
a1
a2
a3
a5
a7
D
E
F
KIPyV NLS peptide
MCPyV NLS peptide
WUPyV NLS peptide
a1
a2
a3
a5
a7
a1
a2
a3
a5
a7
a1
a2
a3
a5
a7
a1
a2
a3
a5
a7
a1
a2
a3
a5
a7
a1
a2
a3
a5
a7

## Slide 5
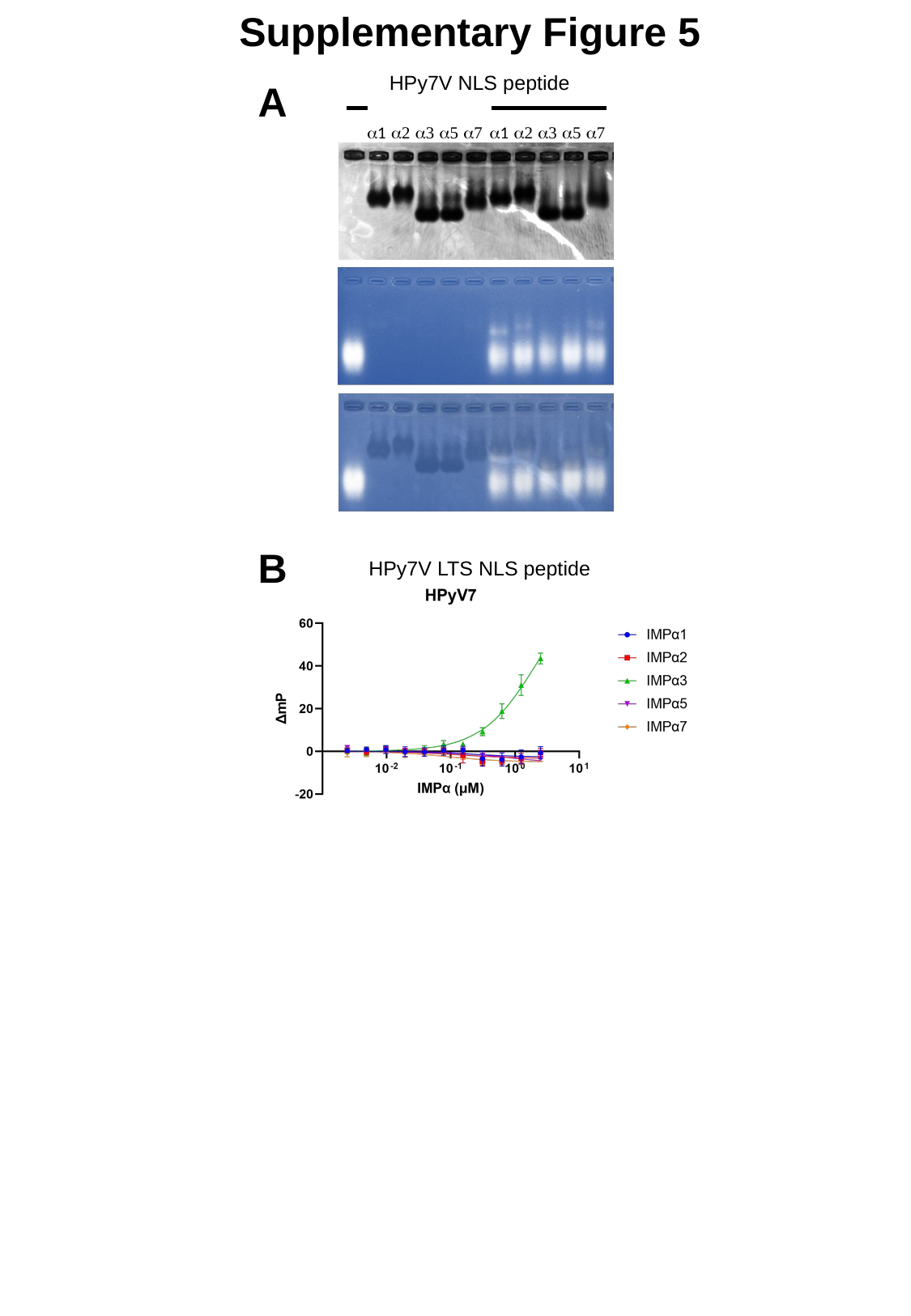

Supplementary Figure 5
A
HPy7V NLS peptide
a1
a2
a3
a5
a7
a1
a2
a3
a5
a7
B
HPy7V LTS NLS peptide

## Slide 6
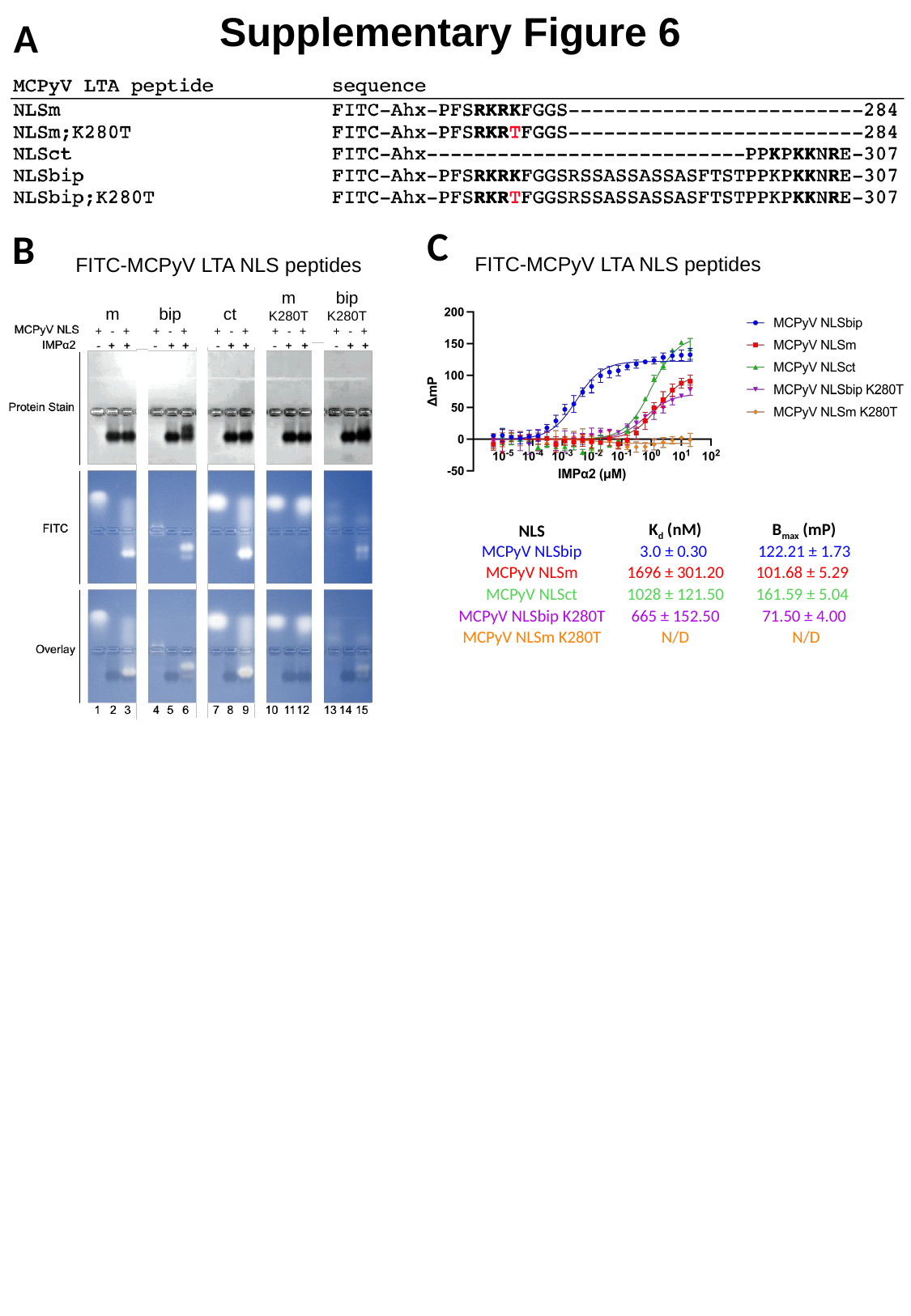

Supplementary Figure 6
A
C
B
FITC-MCPyV LTA NLS peptides
FITC-MCPyV LTA NLS peptides
m
K280T
bip
K280T
m
bip
ct
+ - +
+ - +
+ - +
+ - +
+ - +
| NLS | Kd (nM) | Bmax (mP) |
| --- | --- | --- |
| MCPyV NLSbip | 3.0 ± 0.30 | 122.21 ± 1.73 |
| MCPyV NLSm | 1696 ± 301.20 | 101.68 ± 5.29 |
| MCPyV NLSct | 1028 ± 121.50 | 161.59 ± 5.04 |
| MCPyV NLSbip K280T | 665 ± 152.50 | 71.50 ± 4.00 |
| MCPyV NLSm K280T | N/D | N/D |

## Slide 7
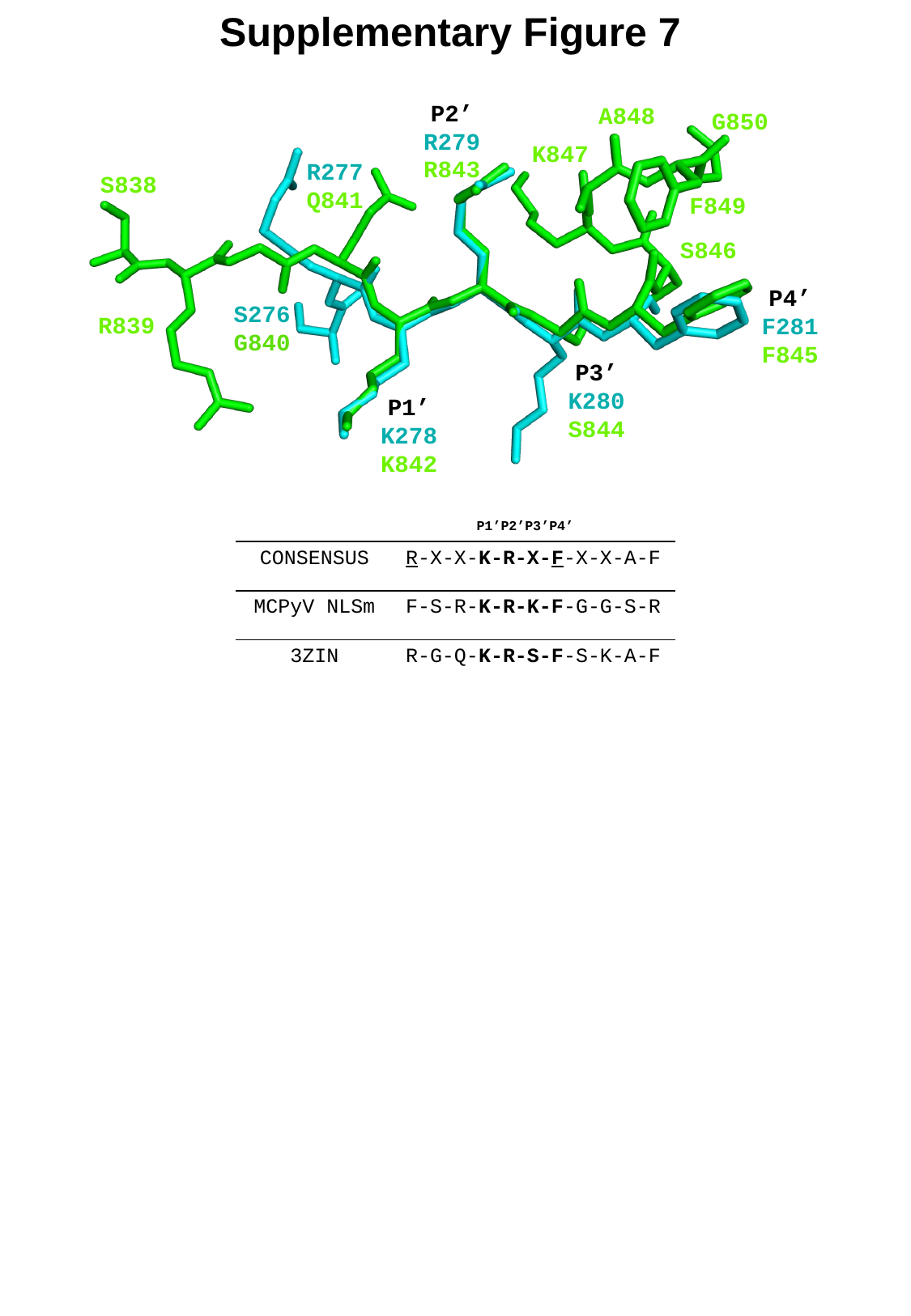

Supplementary Figure 7
P2’
R279
R843
A848
G850
K847
R277
Q841
S838
F849
S846
P4’
F281
F845
S276
G840
R839
P3’
K280
S844
P1’
K278
K842
| | P1’P2’P3’P4’ |
| --- | --- |
| CONSENSUS | R-X-X-K-R-X-F-X-X-A-F |
| MCPyV NLSm | F-S-R-K-R-K-F-G-G-S-R |
| 3ZIN | R-G-Q-K-R-S-F-S-K-A-F |
